# Application of ultrasound-mediated adapalene-coated lysozyme-shelled microbubbles in UVA-induced skin photoaging

**DOI:** 10.1371/journal.pone.0232617

**Published:** 2020-05-21

**Authors:** Ai-Ho Liao, You-Lin Cai, Ho-Chaio Chuang, Cheng-Ying Lee, Yu-Chun Lin, Chien-Ping Chiang

**Affiliations:** 1 Graduate Institute of Biomedical Engineering, National Taiwan University of Science and Technology, Taipei, Taiwan; 2 Department of Medical Engineering, National Defense Medical Center, Taipei, Taiwan; 3 Graduate Institute of Manufacturing Technology, National Taipei University of Technology, Taipei, Taiwan; 4 Department of Pathology, Tri-Service General Hospital, National Defense Medical Center, Taipei, Taiwan; 5 Department of Dermatology, Tri-Service General Hospital, National Defense Medical Center, Taipei, Taiwan; 6 Department of Biochemistry, National Defense Medical Center, Taipei, Taiwan; Argonne National Laboratory, UNITED STATES

## Abstract

Photoaging, the premature aging of skin induced by ultraviolet rays, is characterized by wrinkling, roughness, laxity, and pigmentary changes. Various natural and synthetic retinoids have been explored for the treatment of aging. Among retinoids, adapalene (Ada, 0.3%) is one of the most potent and widely used drugs to treat photoaging. However, it causes irritant reactions that limit its acceptance by patients. Several studies have shown the applicability of Lysozyme (Lys)-shelled microbubbles (MBs) for drug delivery through sonophoresis, and recently we have shown its efficiency to treat inflammatory skin disease. Here, we report the construction of novel Ada-LysMBs based on opposite electric charges for combined effects to treat photoaging. The Ada-LysMBs were self-assembled and had a mean diameter of 2857 nm. The maximum loading efficiency of Ada onto LysMBs was 13.99 ± 0.59%. An acoustic power density of 3 W/cm^2^ for 1 min revealing maximum penetration depth of LysMBs was optimized for further *in vitro* and *in vivo* studies of Ada-LysMBs. It was observed that *in vitro* Ada release from Ada-LysMBs at 6 h after ultrasound (US) treatment was more rapid at pH 7.4 (82%) than at pH 5.5 (73%). Franz diffusion experiments on isolated porcine skin indicated that US approximately doubled Ada delivery by Ada-LysMBs and Ada + LysMBs at 12 h and six-fold Lys permeation by LysMBs at 6 h, compared to these treatments alone. A 5-week *in vivo* study in mice identified significant wrinkle reduction in animals treated with US plus Ada-LysMBs. Our findings indicate that US may be used with Ada-LysMBs in the water phase to treat photoaging by normalizing hyperkeratinization and promoting collagen synthesis.

## Introduction

Ultraviolet (UV) irradiation from the sun has deleterious effects on the human skin and causes sunburn, immune suppression, cancer, and photoaging [1]. UVA (315–400 nm) is a longer wavelength than UVB (280–315 nm) and UVC (100–280 nm) that makes up 95% of sunlight and affects the skin by modifying both epidermis and dermis [2]. Clinically, photoaged skin is characterized by fine and coarse wrinkling, roughness, dryness, laxity, telangiectasia, and solar comedones [3–5]. Pathologically, it is typified by impaired barrier function [6], variable epidermal thickness, dermal elastosis, decreased and fragmented collagen, increased matrix-degrading metalloproteinases, inflammatory infiltrates, and vessel dilation [7]. Such changes have negative effects on the quality of life, with deleterious impact on well-being, physical attractiveness, and self-confidence [8]. Moreover, photoaging correlates with the development of skin cancer, and the incidence of skin cancer is still on the rise despite public education efforts regarding sun protection [9]. Therefore, photoaging represents a common complaint of both cosmetic and medical patients, and it is imperative for clinicians to evaluate new therapeutic approaches.

The ability of topical retinoic acid to improve photoaging changes in the skin as suggested by observations of middle-aged women under treatment with retinoic acid for acne, supported by studies in a hairless mouse model [10–11]. Adapalene (Ada), 6-[3-(1-adamantyl)-4-methoxy-phenyl] naphthalene-2-carboxylic acid (S1 Fig), is a third-generation topical `retinoid prescribed for the treatment of acne (including comedones) and photoaging [12]. Significant improvement in wrinkles, solar lentigines, and other clinical features of photoaged skin with adapalene occur in a dose-dependent manner [13]. Although Ada is more tolerated than other retinoids (e.g., tretinoin), mild to moderate adverse effects such as erythema, scaling, dryness, pruritus, and burning are still expected in 13–45% of patients treated with 0.3% Ada gel [14]. The therapeutic effects are also limited by poor penetration, associated with incompatibility of photosensitization and skin irritation [15]. Thus, strategies designed to enhance Ada permeation may be beneficial for the therapy of photoaged skin.

Recently, the ability of topical carriers such as solid lipid nanoparticles to improve the skin permeability of Ada, thus minimizing its contact with the skin and reducing photosensitization, has been investigated [16]. Sonophoresis using ultrasound (US) combined with drug-loaded microbubbles (MBs) has also been tested *in vivo* to enhance transdermal drug delivery [17–20]. Among these studies, one of our recent studies showed that combined treatment of US and lysozyme (Lys)-shelled MBs significantly reduced the treatment duration and inhibited *Propionibacterium acnes*-induced inflammatory skin diseases [17]. As an antimicrobial peptide, lysozyme has many functions, including antimicrobial properties and anti-tumor and anti-inflammatory functions [21]. Lysozyme has no elastolytic activity *in situ*; nevertheless, it can bind to elastin and limit elastin degradation in sun-exposed skin [22]. Furthermore, egg white lysozyme can promote collagen biosynthesis and extracellular matrix protein production by fibroblasts in the mouse dermis [23].

Taking into account the mentioned background, the hypotheis of this paper is that the ultrasound-mediated adapalen-coated lysozyme-shelled microbubbles may repair the photoaged skin (S2 Fig). In the current research, to enhance the Ada permeation, we constructed novel Ada-coated Lys MBs (Ada-LysMBs) and combined them with sonication by US energy in the water phase. Subsequently, the skin permeation profile was established *in vitro*, followed by validation of its anti-wrinkling effect in a UVA-irradiated hairless mouse model *in vivo*. Histological assessments were performed to demonstrate the recovery of the thickened epidermis and degraded dermis after the application of Ada-LysMBs to the UV-damaged skin.

## Materials and methods

### Ethics statement

This study was carried out in strict accordance with the recommendation in the Guide for the Care and Use of Laboratory Animals of the Latoratory Animal Center (LAC) of National Defese Medical Center. The experimental protocol was approved by the Institutional Animal Care and Use Committee (IACUC, protocol number 16–328) of the National Defense Medical Center. Animals were cared for in compliance with institutional guidelines and regulations (S1 File).

### Preparation and characterization of Ada-LysMBs

**Fig 1:**
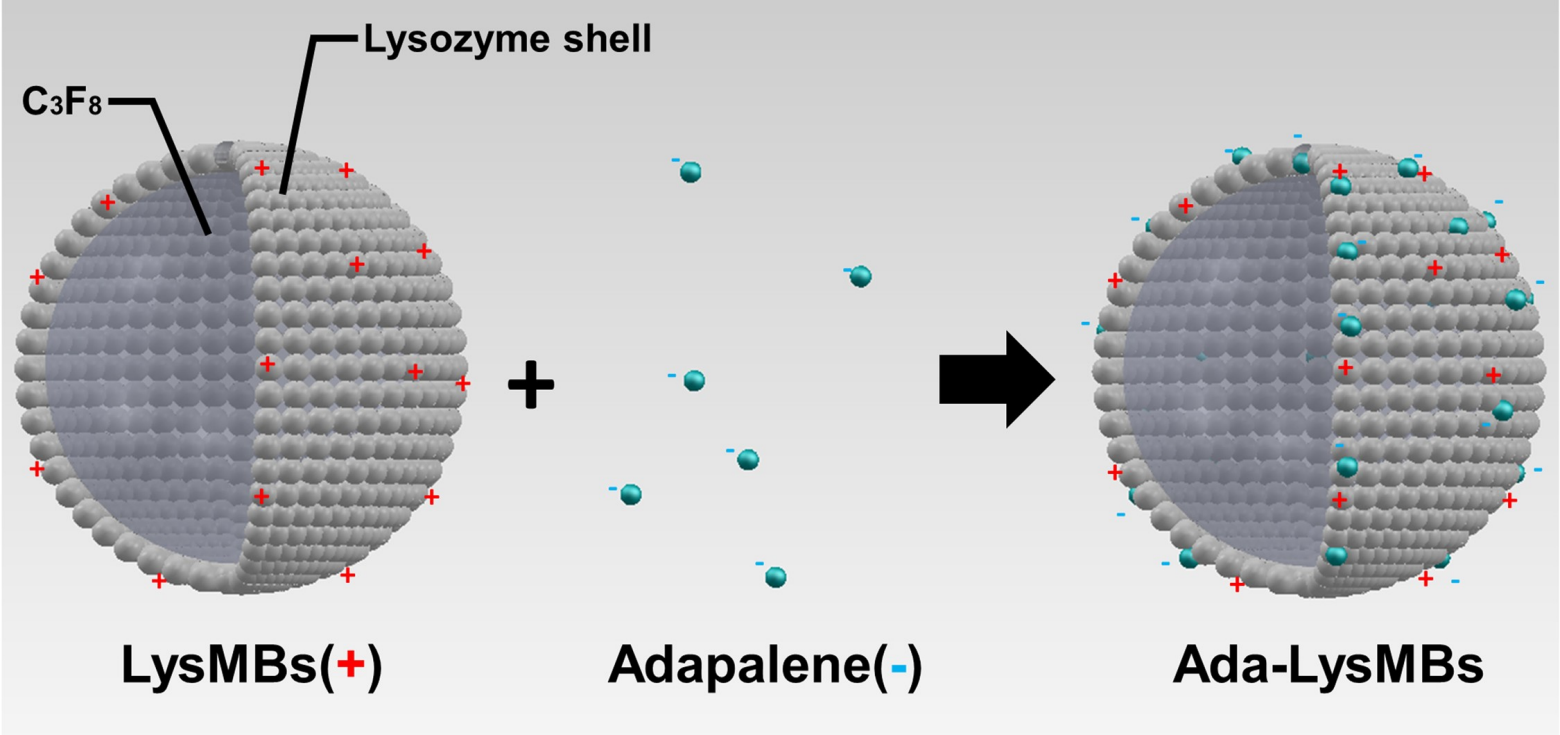
Schematic (not to scale) of the self-assembly of Ada-LysMBs.

Self-assembled Ada-LysMBs were prepared and characterized as shown in Fig 1 and Table 1. Because Lys are positively charged, and its shell has a positive surface potential, it attracts negatively-charged molecules such as Ada [24]. LysMBs were prepared as previously described [17]. To produce Ada-LysMBs, first the solution of Ada (molecular weight = 412.52; Sigma-Aldrich, St. Louis, MO, USA) in dimethyl sulfoxide at different concentrations (0.25, 0.5, or 1 mg/mL) was prepared. The LysMBs were then incubated with 1 ml of Ada solution for 1 h at 4°C on a rotary shaker (50 rpm; Shaker RS-01, TKS, New Taipei City, Taiwan) followed by subsequent washing for three times to remove the unbound, Ada. The number of Ada-LysMBs in the solution was measured using a MultiSizer III device (Beckman Coulter, Fullerton, CA, USA) with a 30-μm aperture probe and a measurement range of 0.6–20 μm. The size distribution in the suspension was measured by dynamic light scattering (Nanoparticle Analyzer, Horiba, Kyoto, Japan). To characterize their morphology, Ada-LysMBs were filtered using a 5-μm syringe filter (Sartorius, Goettingen, Germany) and then hardened using 0.25% glutaraldehyde (Sigma-Aldrich, St. Louis, IL, USA). The morphologies of the hardened Lys-shelled MBs and Ada-LysMBs were studied using scanning electron microscopy at an accelerating voltage of 15 kV after coating the samples with platinum (achieved using 20 mA for 20 min) using an automatic sputter coater (JFC-1300, JEOL, Tokyo, Japan).

**Table 1:** Characterization of Ada and Ada-LysMBs.

		Ada-LysMBs		
Concentration of Ada solution mg/mL)	Ada zeta potential (mV)	Zeta potential (mV)	Size (μm)	Encapsulation efficiency (%)
0	0.5 ± 0.0	65.37 ± 7.65	1.92 ± 0.11	0 ± 0.0
0.25	-12.33 ± 2.36	59.99 ± 0.84	2.25 ± 2.03	4.54 ± 1.4
0.5	-25.67 ± 4.82	56.02 ± 1.08	2.66 ± 0.65	9.22 ± 0.72
1	-34.27 ± 5.79	51.72 ± 4.91	2.81 ± 0.07	13.99 ± 0.59

Data represent the mean ± standard deviation.

### Measurement of penetration depth in agarose phantoms

A model compound, Evans blue (0.1 mg; 960.81 Da; E2129, Sigma-Aldrich, St Louis, MO, USA), was dissolved in 10 mL of phosphate-buffered saline (PBS) and then stirred for 1 h at 4°C. Circular agarose phantoms (0.3%) were constructed with a radius of 1.2 cm and a height of 3 mm (encapsulated with US gel to prevent leakage); the round area of each phantom was loaded with Evans blue or Ada-LysMBs. The probe of the sonoporation gene transfection system (ST 2000V, NepaGene, Ichikawa, Japan) was positioned 3 mm from the top of the phantom. After adding 500 μl of the Ada-LysMBs, the area was sonicated by the 1-MHz US transducer of the sonoporation system successively at the following acoustic power densities: 1 W/cm^2^ for 1 min, 2 W/cm^2^ for 1 min, and 3 W/cm^2^ for 1 min. The duty cycle was set at 50%, and a 1.2-cm-diameter transducer was used. The change in temperature during US sonication at power densities of 2 W/cm^2^ and 3 W/cm^2^ for 1 min in 37°C did not exceed 0.3°C, as measured by a thermometer (Optris LS, Optris, Berlin, Germany) [19].

The Ada-LysMBs were subsequently removed from the surface and the area was washed three times for 1 min each with PBS. The Evans blue solution was then loaded onto the phantom and incubated for 1 min prior to its removal and washed thrice for 1 min each with PBS. Sections (3 mm thick) of the phantom were cut and prepared for evaluation using light microscopy. The penetration depth of the Evans blue was measured using MATLAB (The MathWorks, Natick, MA, USA). The light microscopy images were converted into 8-bit grayscale images and image histogram-based binarization was performed using peak-and-valley thresholding of the target based on data from multiple experiments [25]. The boundary was then detected using Sobel operator-based edge detection [26], using the same threshold to process all the images. Finally, the area of the penetration region was measured and divided by the length of the *x*-axis of the image to obtain the mean penetration depth (*y*-axis) of the Evans blue.

### *In vitro* release study

The *in vitro* release behavior of Ada from LysMB vesicles was investigated using a dialysis method. A suspension of Ada-LysMBs (1 mL of the original concentration after production) was loaded into a dialysis bag with a molecular weight cut-off of 12–14 kDa and dialyzed against the release media (PBS) at pH values of 5.5 and 7.4 within 37 ± 0.5°C, with stirring at 600 rpm by a magnetic bar. After 0.5 h, the 1-MHz unfocused US therapy transducer (ST 2000V; NepaGene) was positioned 3 mm from the top of the dialysis bag under the liquid level, and sonication was applied at 3 W/cm^2^ (acoustic pressure = 0.266 MPa) for 1 min (S3 Fig). Samples (1 mL) were taken from the release medium after 0.1, 0.2, 0.3, 0.4, 0.5, 1, 2, 3, 4, 5, and 6 h and replaced by the same volume of PBS. These samples were stored in a freezer until analysis using a UV-visible spectrophotometer (Lambda 40; Perkin Elmer, Bridgeville, PA, USA). The mean values obtained from four replicates were calculated. The drug release profile of Ada was examined as a control. The cumulative release percentage of Ada from Ada-LysMBs was calculated using the following equation [27]:

$$R=\frac{c_{n}v_{0}+\sum_{i=0}^{n-1}c_{i}v_{i}}{W}\times 100\%$$

where *R* was the release rate, *c*_*n*_ was the drug concentration in the release medium each time interval, *v*_0_ was the total volume of the release medium (100 mL), *v*_*i*_ was the volume of medium withdrawn at each time point (1 mL), *c*_*i*_ was the drug concentration in the release medium at time intervals, and *W* was the mass of drug employed [27].

### *In vitro* skin penetration by Ada and Lys

Fresh porcine ear skin was obtained from the Affiliated Slaughterhouse of New Taipei City Meat Market, and all associated experiments were completed within 6 h. A 2-mm-thick sample of pigskin was harvested using a Humby knife, carefully cleaned with PBS, and cut into square pieces (2 cm × 2 cm). Skin penetration was then tested using static Franz diffusion cells over an area of 2.14 cm^2^ with the experimental design described in our previous study (Liao et al., 2016b). The temperature of the diffusion assembly was maintained at 37°C. Samples (250 μL) of the receptor solution were taken at 0.5, 1, 2, 3, 4, 5, 6, 8, and 12 h, and replaced by the same volume of fresh receptor solution. After 0.5 h, sonication was applied for 1 min at 3 W/cm^2^ (acoustic pressure = 0.266 MPa) using the 1-MHz ultrasound transducer (ST 2000V; NepaGene) positioned 3 mm from the top of the skin (S4 Fig). Samples were kept in a freezer until they were analyzed using a UV-visible spectrophotometer (Lambda 40, Perkin Elmer, Waltham, MA, USA) for Ada concentration or a fluorometric lysozyme activity assay kit (BioVision K236, Milpitas, CA, USA) for Lys activity.

At the end of the penetration experiments (i.e., after 12 h), the skin sample was detached from the diffusion cell and carefully rinsed five times with distilled water to remove excess Ada and Lys from its surface. For the Ada assay, the skin was cut into 0.1 g pieces and homogenized with 2 mL of receptor solution for 2 min at 10,000 rpm (Polytron-Aggregate PT3100, Kinematica, Luzern, Switzerland). The homogenized suspension was centrifuged twice for 25 min at 3,100 *g* (Thermo Fisher Scientific, Bremen, Germany). Subsequently, 200 μL of the supernatant were used for estimation of the concentrations of Ada using a UV-visible spectrophotometer, and an Ada standard calibration curve. For the Lys test, we repeated another 3 samples over 6 h, and the tested skin was cut into 0.1 g pieces, homogenized with 1 mL of ice-cold lysozyme assay buffer containing protease inhibitor, and kept on ice for 10 min. The samples were centrifuged at 12,000 g at 4°C for 5 min, and the supernatant was collected. Then, 40 μl of the sample was added into desired wells in a white 96-well plate. The fluorescence intensity was measured at Ex/Em = 360/445 nm at 37°C with an endpoint setting using an Epoch Microplate Spectrophotometer (Biotek, Winooski, VT, USA). A Lys standard calibration curve was prepared according to the manufacturer’s instructions to determine the sample concentrations of Lys.

### Animal treatments

A mouse model of photoaging was established using 6-week-old female BALB/c nude mice weighing 18–20 g [28] that were obtained from Bio Lasco (Taipei, Taiwan). All animals were housed in the laboratory animal center of National Defense Medical Center at a constant temperature of 22 ± 2°C, humidity of 55–70%, and a controlled 12 h light–12 h dark cycle according to institutional guidelines. Once weekly, home cages were cleaned and equipped with new bedding by a professional animal keeper. Animals were fed with complete pellet diet and had free access to food and water at all times. The diet intake and activity of each animal was monitored every day. The animals were acclimatized for 7 days before the experiments. Rectangular cast-iron support with three forks, a universal clamp with holder, and a stainless steel clamp was used to fix two UV lamps as the UVA irradiation sources (S5). These were 18 cm apart and delivered 0.606 mW/cm^2^ for 60 min/day at wavelengths between 350 nm and 390 nm, at a distance of 10 cm from the backs of the mice. Before the BALB/c nude mice study, the photoaging model was established after general anesthesia by isoflurane inhalation (2% in oxygen). The experimental procedure was described in S6 Fig. After 18 days of irradiation for 28 days, each mouse had received a total dose of 39 J/cm^2^ [29]. Total number of animals used in this study was thirty. In addition to one non-irradiation (normal) group (*n* = 5), the UVA-irradiated animals were divided into the following five groups (*n* = 5 per group), and the following treatments were applied once daily for 5 weeks: (i) no treatment (control); (ii) 1 mL of Ada at 35 μg/mL only (Ada); (iii) US plus 1 mL of Ada at 35 μg/mL (US + Ada); (iv) US plus 1 mL of Ada at 35 μg/mL plus 1 mL LysMBs (US + LysMBs + Ada); and (v) US plus Ada-LysMBs containing 35 μg/mL Ada and 3.55 × 10^7^ MBs/mL (US + Ada-LysMBs). The US was applied at 3 W/cm^2^ (acoustic pressure = 0.266 MPa) for 1 min. The treatments were applied once daily for 5 weeks, and the irradiated area was recorded using a monocular camera.

### Image processing for wrinkle measurement

The number of wrinkles was calculated using MATLAB on each measurement day before and after treatment. The images were converted from color to grayscale before performing histogram-based binarization [30]. This histogram-based binarization employed peak-and-valley thresholding based on the histogram representing multiple experiments [26]. The boundary was then detected by Canny edge detection [31], using the same threshold when processing all of the images. Finally, the numbers of pixels relating to wrinkles enclosed by the leading edge were calculated (S7 Fig). The healing rate was then calculated using the following equation [32]:

$$\text{Recovery}\;\text{rate}\left(\%\right)=\frac{W_{1}-W_{n}}{W_{1}}\times 100\%$$

Where *W*_1_ is the number of wrinkles immediately after UVA treatments, and *W*_*n*_ is the wrinkle area at each measurement time point.

### Histopathology and histochemistry

After the UV irradiation experiments, on day 21, the tested mice were euthanized by CO_2_ overdose and the hairless skin samples (approximately 8 mm × 8 mm) were cut immediately from the treatment area and stored in a 10% formalin solution. The specimens were then embedded in paraffin. Tissue sections (4-mm thick) were deparaffinized, rehydrated, and washed twice in the buffer. They were then stained using hematoxylin & eosin (H&E) or a modified Verhoeff van Gieson (VVG)-based elastic-stain kit (ScyTek Laboratories, Inc., Cache, UT, USA) according to the manufacturers’ recommended protocols. The histopathological and histochemical data were interpreted by a dermatologist (Chiang, C.P.) and a pathologist (Lin, Y.C.). The mean epidermal and dermal thicknesses identified using H&E was measured in four different fields of the same slide.

### Statistical analysis

The data were analyzed using Student’s *t*-test. *P* < 0.05 was considered indicative of a significant difference. Results were also expressed as the mean ± standard deviation. The statistical significance of skin thickness differences among the six *in vivo* study groups was evaluated by one-way analysis of variance and the Bonferroni post-hoc test.

## Results

### Characterization of Ada-LysMBs

**Fig 2:**
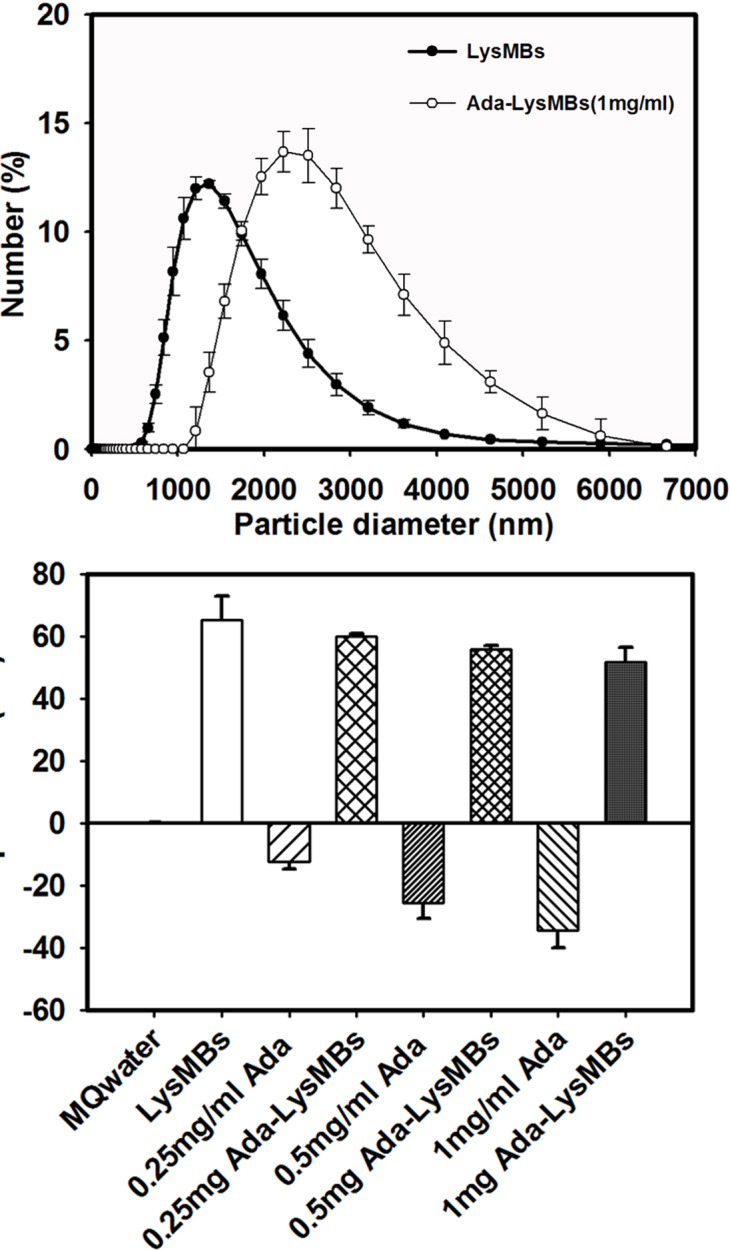
Quantification of the size distributions (A) and zeta potentials (B) of LysMBs and Ada-LysMBs.

The diameter of the `LysMBs and Ada-LysMBs was estimated to be 1975 ± 124 nm and 2857 ± 741 nm, respectively (Fig 2A), while the concentration of LysMBs was 1.197 ± 0.196 × 10^8^/mL (*n* = 3). Our previous study indicated that there were 25 mg/mL Lys in the original solution of Lys-shelled MBs constructed using a sonicator power of 120 W [17]. The zeta potential of the LysMBs and Ada-LysMBs, dispersed in an aqueous solution (pH = 6.4, resistance = 18.2 mΩ), was measured using a Nanoparticle Analyzer (Horiba). Lys is a positively charged protein at pH 7, and the LysMBs had a potential of +65.37 ± 7.65 mV. The potentials of 0.25, 0.5, and 1 mg/mL Ada were −12.33 ± 2.36, −25.67 ± 4.82, and −34.27 ± 5.79 mV. The surface potentials of 0.25 mg of Ada-LysMBs (0.25 mg/mL Ada), 0.5 mg of Ada-LysMBs (0.5 mg/mL Ada), and 1 mg of Ada-LysMBs (1 mg/mL Ada) were +59.99 ± 0.84, +56.02 ± 1.08, and +51.72 ± 4.91 mV, respectively (Fig 2B and Table 1) (*n* = 6).

**Fig 3:**
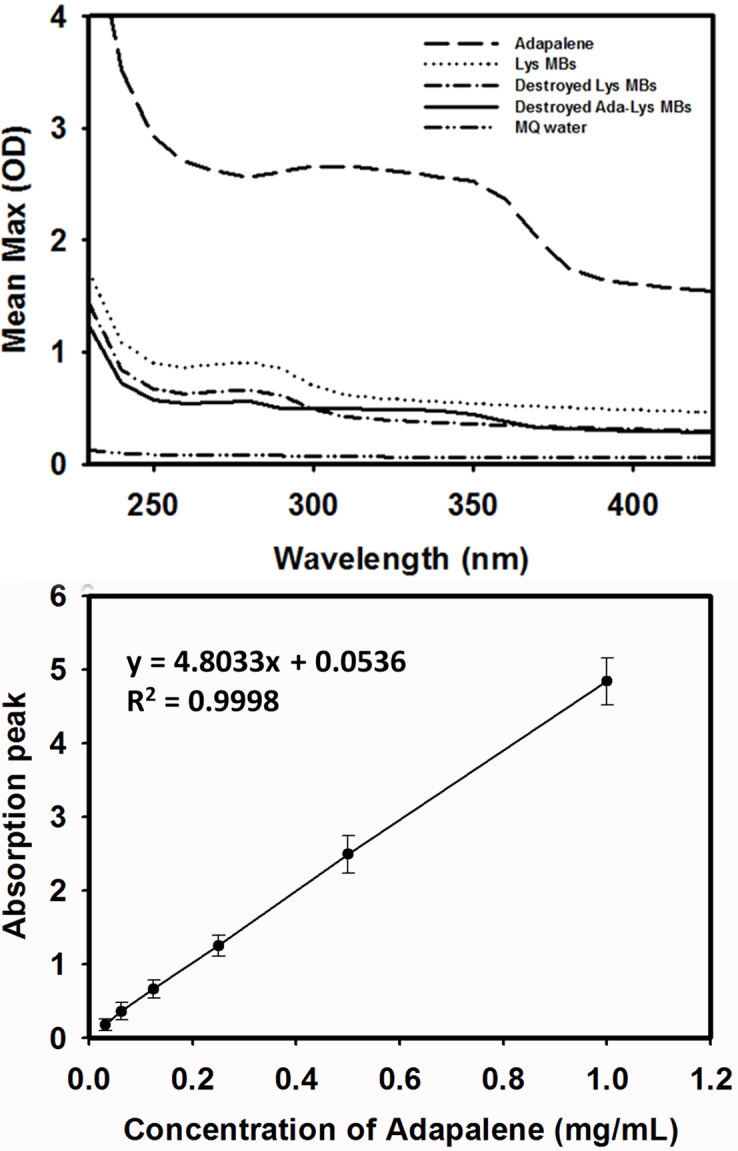
(A) Absorbance spectra of the indicated solutions, (B) Ada standard calibration curve used to determine the concentrations of samples.

**Fig 4:**
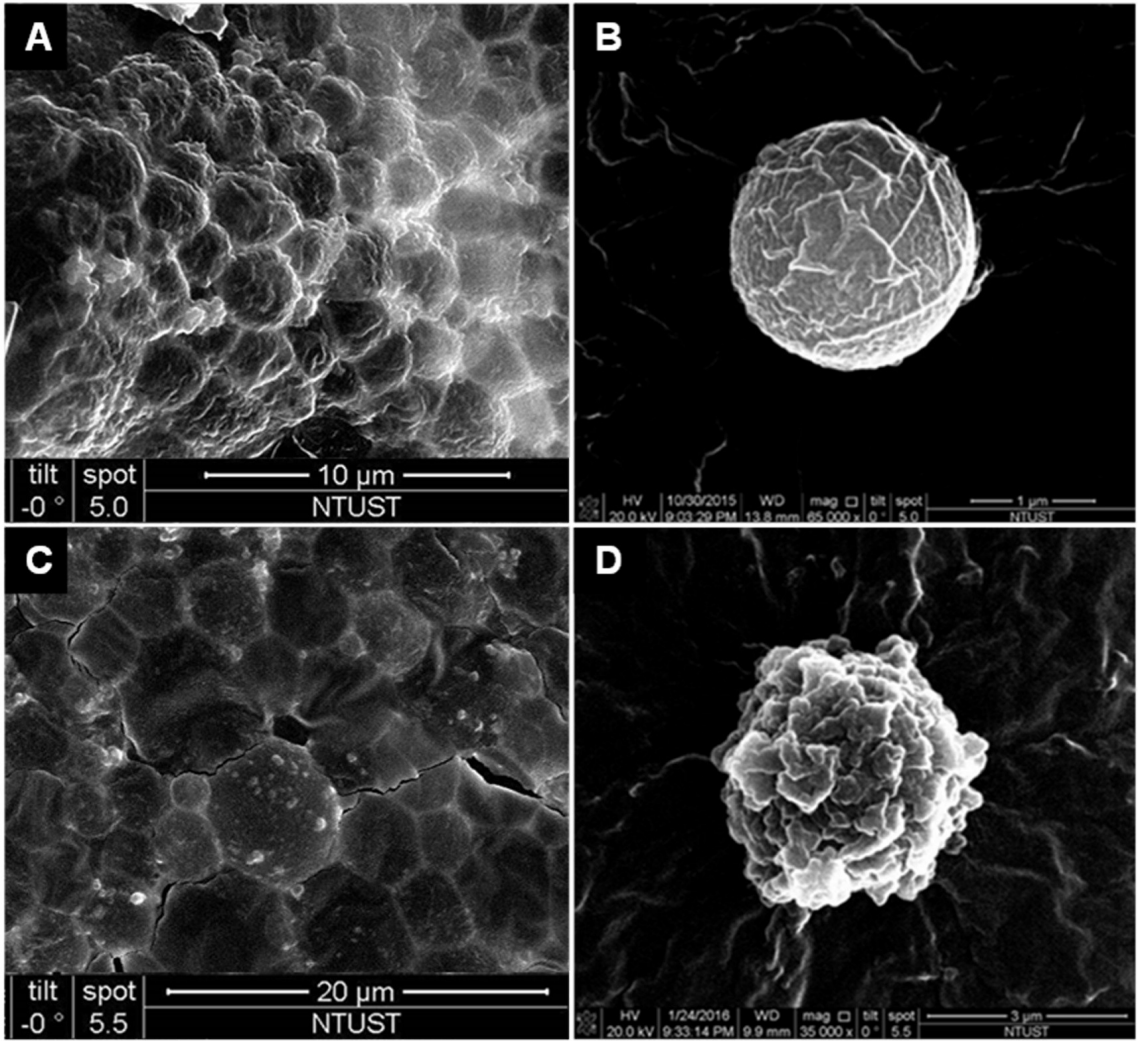
Scanning electron microscopy images of the LysMBs and Ada- LysMBs. (A, C) Multiple LysMBs and Ada-LysMBs, (B, D) single LysMB, and Ada-LysMBs.

The absorbance spectra of LysMBs and Ada-LysMBs following destruction by sonication and of Ada solution, LysMBs, and Milli-Q water are shown in Fig 3A. LysMBs absorbed light at 280 nm, whereas Ada and filtered Ada-LysMBs following sonication absorbed light at 321 nm. Fig 3B shows the Ada calibration curve. Ada encapsulation efficiency (Table 1) was analyzed using a UV-visible spectrophotometer, revealing a maximum loading efficiency of 13.99 ± 0.50% (*n* = 6) for Ada-LysMBs. These Ada-LysMBs were used for the subsequent *in vitro* release study and the *in vivo* experiments. Fig 4 shows scanning electron microscopy images of multiple and single LysMB (A, B) and Ada-LysMB (C, D). Compared to a single LysMB (Fig 4B), the morphology of Ada-LysMB (Fig 4D) was observed to be different, and some nanoscale particles coated on the LysMB shell were observable.

### Penetration depth in agarose phantoms

**Fig 5:**
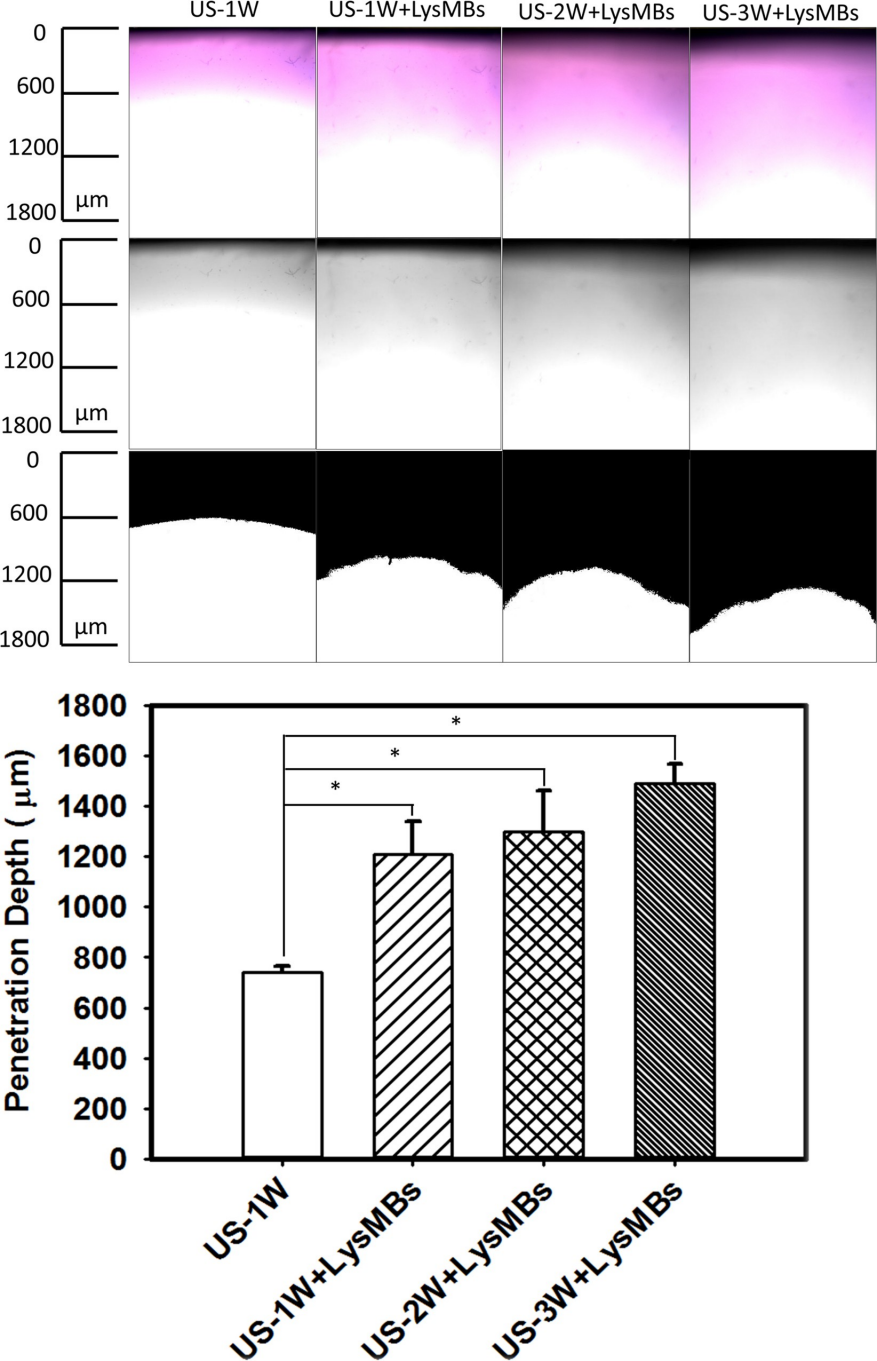
(A) Light microscope evaluation of agarose phantoms that had been treated as indicated. (B) Quantification of the penetration depth of Evans blue. Data represent the mean values and standard deviations. *p < 0.05.

The microscopy images of the agarose phantoms were obtained before (upper row) and after (middle and lower rows) MATLAB-based image processing for combining US at 1 W/cm^2^, 2 W/cm^2^, and 3 W/cm^2^ with LysMBs (n = 4) after Evans blue solution was allowed to stand for 1 min (Fig 5A). Subsequently, the penetration depths in various groups were quantified (Fig 5B). The penetration depth using LysMBs was observed to be higher when US was applied at 3 W/cm^2^ (1488.43 ± 79.43 μm), as compared to 1 W/cm^2^ (1208.5 ± 132.06 μm) and 2 W/cm^2^ (1297.18 ± 163.47 μm) (*p <* 0.05). Based on the results, we have chosen a power density of 3 W/cm^2^ for subsequent *in vitro* and *in vivo* experiments.

### *In vitro* release study

**Fig 6:**
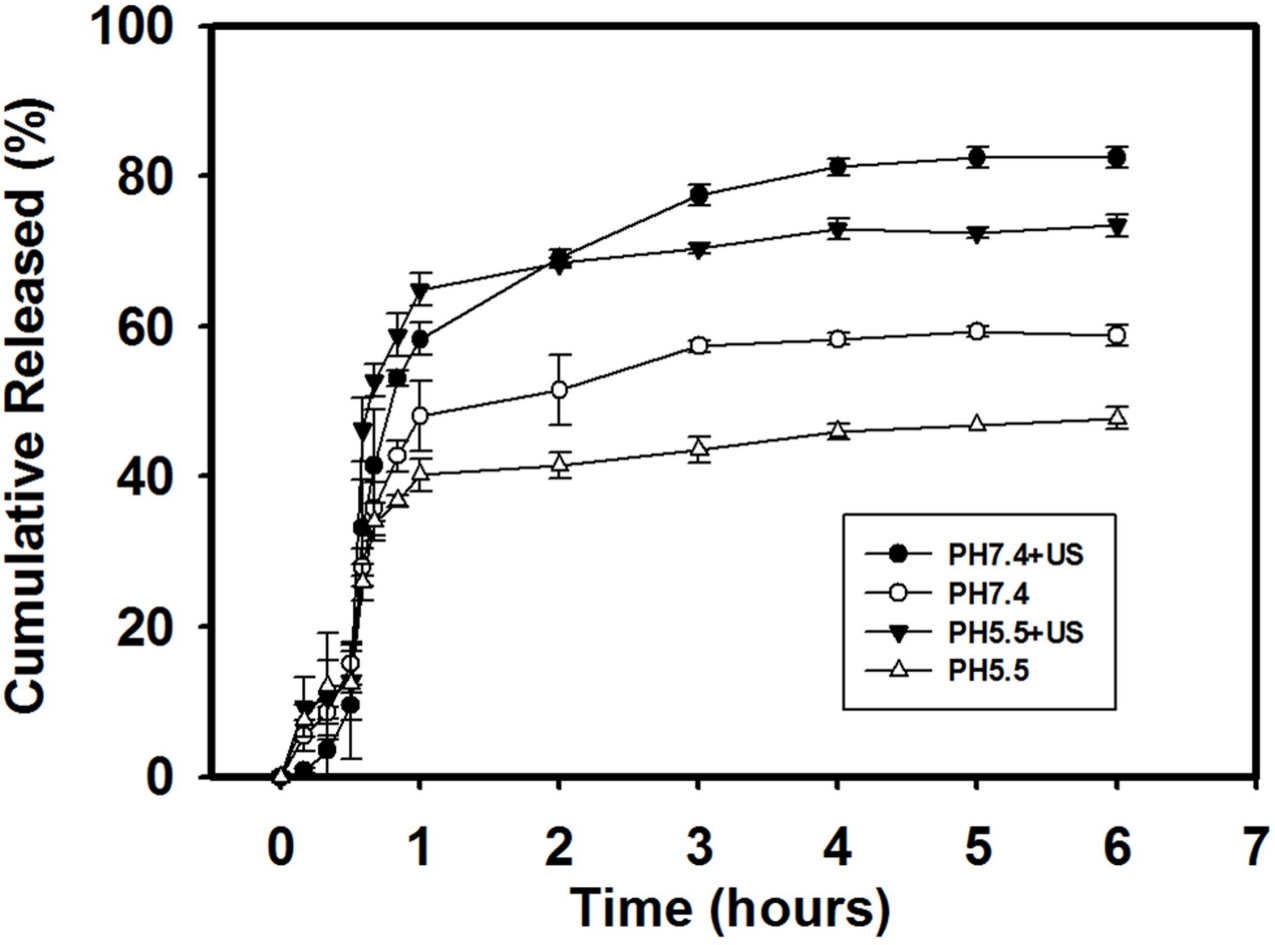
Cumulative Ada release from Ada-LysMBs after 6 h with and without ultrasound sonication in PBS at the indicated pH.

The effect of the US on the cumulative Ada release from Ada-LysMBs over 6 h in PBS (pH 5.5 and 7.4) is shown in Fig 6. In the pH 5.5 environment, with US sonication, 46.3% of Ada diffused through the dialysis membrane (relative to the control) over the first 0.5833 h (i.e., 35 min), which was found to be increased to 73.4% after 6 h. In the pH 7.4 environment, the diffusion of free Ada through the dialysis membrane relative to the control was only 33.2% at 0.5833 h and 82.4% after 6 h. In the absence of sonication, 47.8% of Ada had crossed the dialysis membrane within 6 h at pH 5.5, and 58.8% at pH 7.4. With sonication, the *in vitro* release profile of Ada showed a rapid release from Ada-LysMBs during the first 2 h up to 68.4%, and 69.2% at pH 5.5 and pH 7.4, respectively, followed by a slower but sustained release up to 73.4% at pH 5.5 and 82.4% at pH 7.4 after 6 h. These findings indicated that ultrasound energy enhanced the drug release by 40.1–260.9%, and also demonstrated that the efficiency of Ada release from Ada-LysMBs was affected by the pH of the release medium.

### *In vitro* skin penetration by Ada and Lys

**Fig 7:**
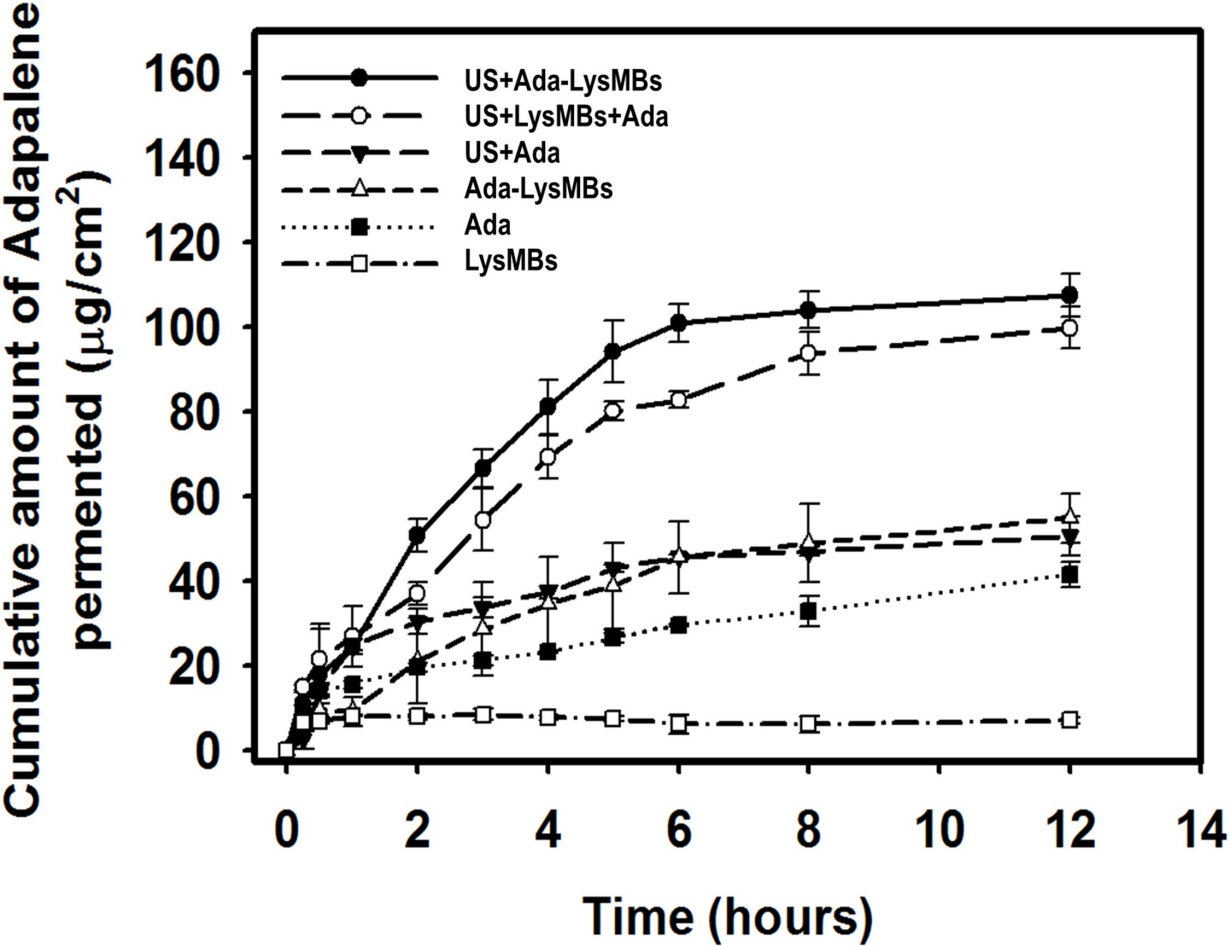
Percutaneous penetration of Ada in isolated skin treated as indicated for 12 h analyzed using a UV-visible spectrophotometer. Data represent the mean values and standard deviations.

Fig 7 shows the percutaneous penetration of Ada over 12 h, as analyzed using a UV-visible spectrophotometer. The concentration of Ada that crossed the skin in the US + Ada-LysMBs group increased rapidly to 94.3 ± 2.3 μg/mL during the first 5 h and then gradually leveled off between 6 and 12 h. At 12 h, a significantly greater amount of Ada crossed the skin in the US + Ada-LysMBs group (107.5 ± 5.1 μg/mL), as compared with the US + LysMBs + Ada (99.9 ± 4.9 μg/mL), US + Ada (50.7 ± 4.5 μg/mL), Ada (41.7 ± 3.0 μg/mL), Ada-LysMB (54.9 ± 5.8 μg/mL), and LysMB (7.2 ± 0.8 μg/mL) groups (*p <* 0.05). The use of the US resulted in a 2.0- and 2.4-fold increase in the Ada penetration from US +Ada-LysMBs, and US + LysMBs + Ada, respectively. Table 2 indicates that the amounts of Ada deposited in the skin appeared to be higher in the US + Ada group than in the Ada group and were significantly higher when compared to the Ada-LysMB, US + LysMB + Ada, and US + Ada-LysMB groups. In addition, the percutaneous penetration of Lys over 6 h was evaluated by a fluorometric lysozyme activity assay. The concentration of permeated Lys in US + LysMB group (66.03 ± 9.05 U/mL) was significantly higher than that in the LysMB group (11.50 ± 4.96 U/mL) over 6 h (*p <* 0.001). The amount of Lys deposited in the skin was also higher in the US + LysMB group (37.65 ± 6.03 U/mL) than in the LysMB group (28.32 ± 1.90 U/mL).

**Table 2:** Levels of Ada detected in the indicated compartments at 12 h.

Group	Weight (g)	Amount in skin (μg/mL)	After 12 h under skin (μg/mL)	Amount in and under skin (μg/mL)
LysMBs	0.1005 ± 0.0003	22.87 ± 2.20	7.18 ± 0.63	30.05 ± 2.83
Ada	0.1005 ± 0.0011	48.49 ± 3.60	41.71 ± 3.01	90.2 ± 6.61
Ada-LysMBs	0.1037 ± 0.005	27.03 ± 1.31	54.94 ± 5.81	81.97 ± 7.12
US + Ada	0.1015 ± 0.0002	58.07 ± 1.48	50.71 ± 4.53	113.01 ± 6.01
US + LysMBs + Ada	0.1026 ± 0.0024	35.72 ± 4.73	99.86 ± 4.9	135.58 ± 9.63
US + Ada-LysMBs	0.1003 ± 0.0018	31.86 ± 1.74	107.5 ± 5.11	139.36 ± 6.85

Data represent the mean ± standard deviation.

### Animal treatments

**Fig 8:**
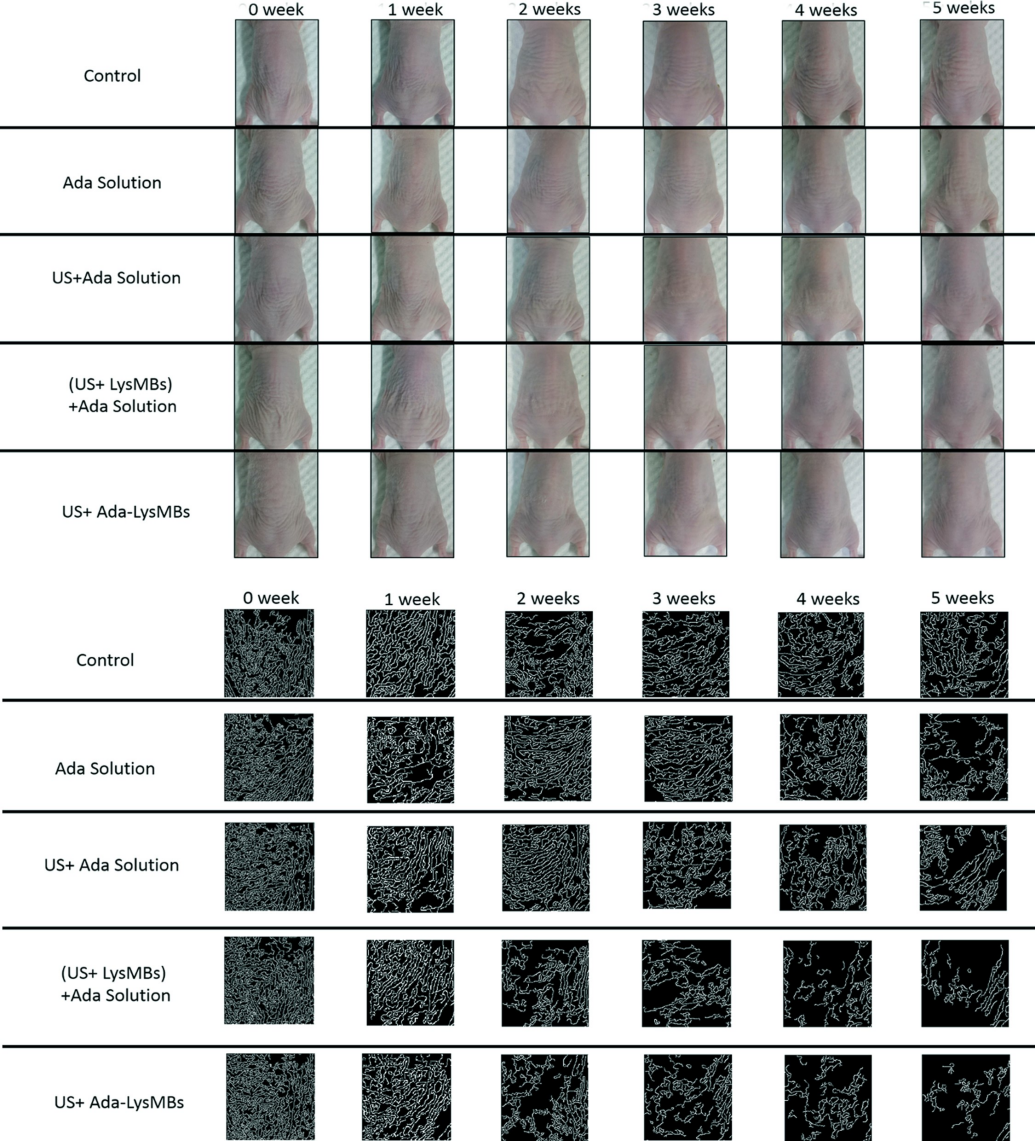
Photographs (A) and Canny wrinkle detection (B) of mice treated as indicated are shown at various time points during the treatment.

**Fig 9:**
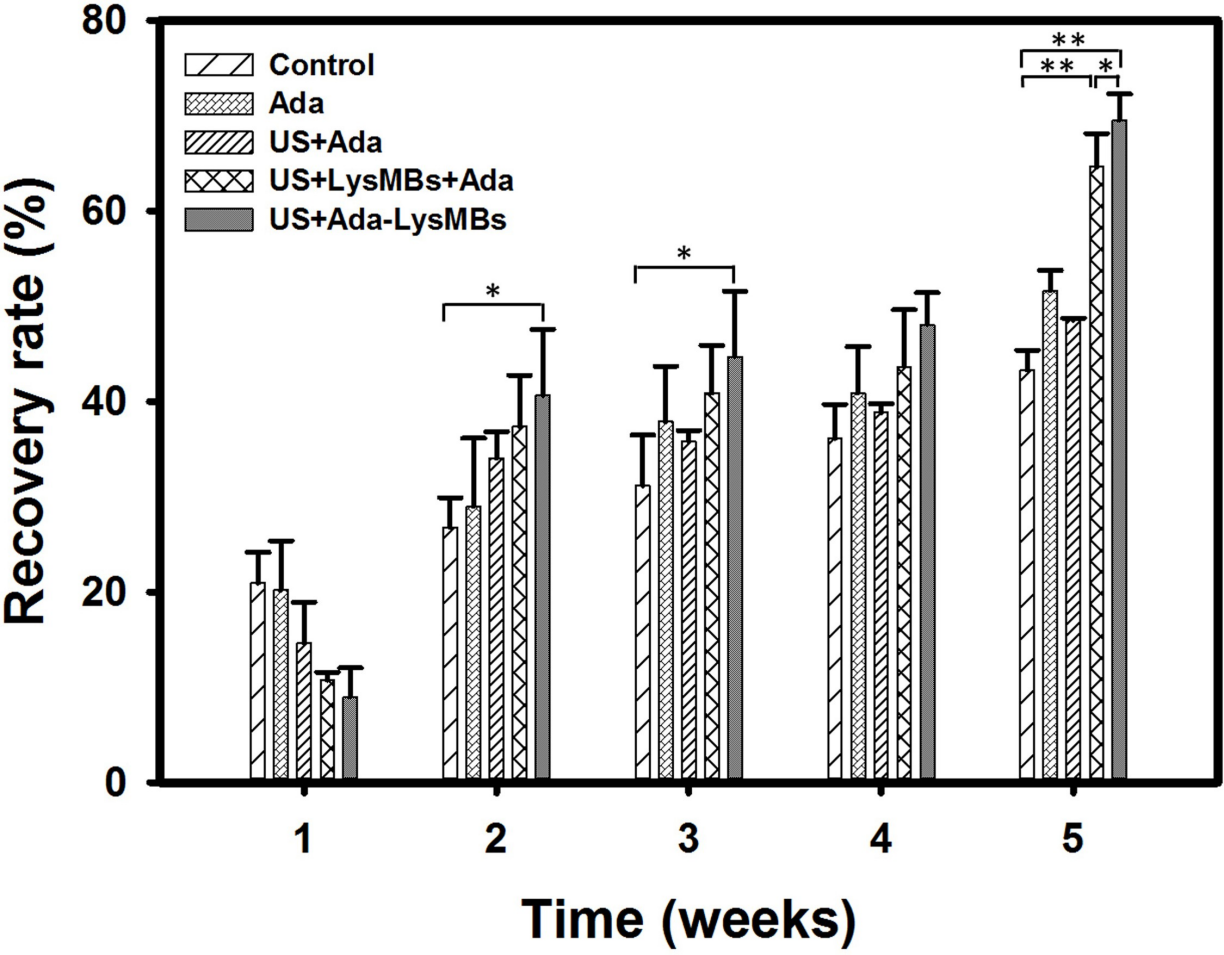
The recovery rate of photoaged skin of mice. The wrinkle areas of the mice were calculated using the detected edge, and the Canny method in MATLAB. *p < 0.05 ** *p*< 0.01.

The UVA-irradiated hairless mice were divided into five groups (*n* = 5 per group) as mentioned in Materials and Methods. Fig 8A shows photographs of mouse skin wrinkles before (day 0) and after UVA irradiation and treatment as indicated (control, Ada, US + Ada, US + LysMBs + Ada, US + Ada-LysMBs) at various time points. Fig 8B shows the wrinkle areas identified using the Canny method in MATLAB. Fig 9 shows that the recovery rate in the US + Ada-LysMB group was increased (*p* < 0.05) in the second and third weeks as compared with the other groups. In the fourth week, the recovery rate of the US + Ada-LysMB group (48.06 ± 3.33%) was close to that of the US + LysMB + Ada group (43.66 ± 5.98%); both were greater than the rates observed in the control (36.11 ± 3.59%), Ada (40.88 ± 4.88%), and US + Ada (38.83 ± 0.93%) groups. In the fifth week, there was a significant difference (*p* < 0.05) between the US + Ada-LysMB group (69.53 ± 2.77%) and the US + LysMB + Ada group (64.63 ± 3.51%). At this time, the recovery rate in these two groups remained higher (*p* < 0.01) than that of the other three groups. No important adverse events in each experimental group during the animal treatments.

### Histopathology and histochemistry

**Fig 10:**
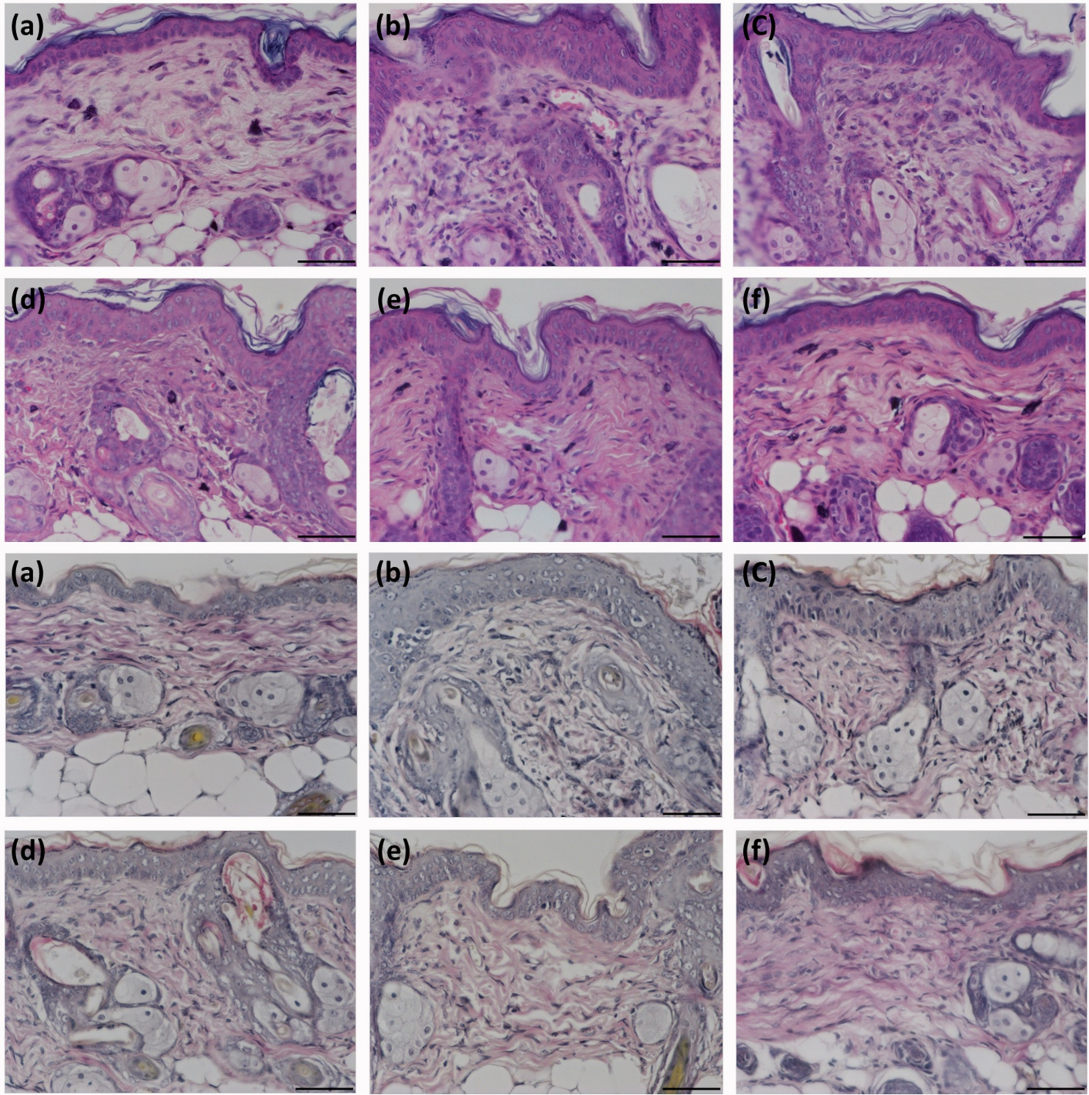
Representative samples showing UV irradiation-induced morphological changes in mouse skin. The upper panel (A) shows hematoxylin and eosin staining, and the lower panel (B) shows modified VVG staining. The sub-panels show samples from a non-exposed normal mouse (a), a UV-irradiated control mouse (b), and UV-irradiated mice treated with Ada (c), US + Ada (d), US + LysMBs + Ada (e), and US + Ada-LysMBs (f). Scale bar = 50 μm; original magnification 400 ×.

The non-UV-irradiated mice showed normal skin with a very thin epidermis that consisted of 2–3 cell layers (Fig 10A/a). The dermis showed even collagen bundles that were stained red, and the thin and sparse elastic fibers were stained black by the modified VVG method (Fig 10B/a). After UV irradiation, photoaging changes with a rough epidermis, an increased number of hyperplastic fibroblasts, and vessel ectasia developed (Fig 10A/b). In addition, the epidermal and dermal thicknesses increased by approximately 2 fold (Table 3). However, treatment with US + LysMBs + Ada or US + Ada-LysMBs significantly attenuated the epidermal thickness, with the US + Ada-LysMB group nearly matching the non-UV-irradiated normal group. UV exposure decreased the amount of collagen (Fig 10B/b), whereas treatment with US + Ada-LysMBs ameliorated this loss (Fig 10B/f). In addition, UV irradiation caused fragmentation and tangling of the elastic fibers (Fig 10B/b). Treatment with US + Ada-LysMBs also resulted in the recovery of these elastic fibers to the levels observed in non-exposed normal mice (Fig 10B/f).

**Table 3:** Epidermal and dermal thickness in BALB/c mice at the end of the *in vivo* wrinkle experiment.

Groups	Epidermal thickness (μm)	Dermal thickness (μm)
Normal	11.50 ± 0.55	68.50 ± 4.89
Control	19.83 ± 0.98*	84.17 ± 7.81**
Ada	18.17 ± 1.60*	79.0 ± 11.17
US + Ada	17.0 ± 2.0*	80.0 ± 7.46
US + Ada + LysMBs	13.67 ± 1.63	72.33 ± 5.57
US + Ada-LysMBs	12.0 ± 0.63	69.83 ± 6.52

Data represent the mean ± standard deviation. The control group was exposed to UV irradiation only. *p < 0.001 **p < 0.05 as compared to the normal group (not exposed to UV irradiation); Bonferroni test.

## Discussion

Photoaging, in addition to being a cosmetic problem with psychosocial consequences, correlates with the development of skin cancer and is characterized by coarse wrinkling, laxity, dry, rough texture, and uneven pigmentation [9]. Topical retinoids are effective in treating clinical signs of photoaging, but their potential for irritation has prompted clinicians to switch to less irritating agents [5]. In the present study, we found that Ada-LysMBs could effectively deliver retinol and lysozyme to photodamaged mouse skin and achieve significant anti-wrinkle effects. The unique Ada-LysMBs were assembled using the electrostatic attraction between Ada (negatively charged) and LysMBs (positively charged) in Milli-Q water [33]. This study found that the preparation of Ada-LysMBs in the presence of higher amounts of Ada increased Ada encapsulation (Table 1), with 1 mg/mL Ada producing the highest loading efficiency (13.99 ± 0.59%). The final Ada concentration was approximately 0.014%, which was much lower than that in the 0.3% Ada gel used in photoaging treatments; therefore, our treatment might cause less irritation. Indeed, no visible contact dermatitis was noted after applying Ada-LysMBs during our animal experiments.

Moreover, Ada-LysMBs were stable in Milli-Q water and could, therefore, be used in combination with ultrasound to enhance the skin penetration of Ada. The *in vitro* investigation found that the use of ultrasound enhanced Ada release by 24–26%; this depended on the pH, which altered the efficiency of Ada release from Ada-LysMBs. The higher *in vitro* release of Ada was observed at pH 7.4 than at pH 5.5. At a low pH, Lys are highly positively charged, and this may increase the stability of the interaction between Ada and LysMBs, thus inhibiting Ada release from Ada-LysMBs [34–35]. Because the pH value from the mouse skin surface beneath the viable epidermis increased from 5.9 to 7 [36], there could be a controlled-release effect of Ada-LysMBs during US-assisted permeation into the deeper epidermis.

The *in vitro* permeation profiles of Ada through the skin are shown in Fig 7 and Table 2. These findings indicate that the Ada concentration in the Franz cell receptor chamber increased steadily over time. Although this increase occurred more rapidly in the US + Ada-LysMB and US + LysMB + Ada groups during the first 5 h, relative to the other groups, it gradually leveled off between 6 and 12 h. Our results indicate that the combination of the US with Ada-LysMBs or LysMBs + Ada either increased the total amount of Ada delivered or influenced the time profile of Ada delivery as compared to Ada and US + Ada groups. The combination of ultrasound and Ada-LysMBs significantly improved the permeation of Ada, as compared with the other treatment groups. LysMBs might provide a new topical carrier that can enhance Ada skin permeation without using any potentially irritant enhancers in commercial Ada gel (e.g., poloxamer or propylene glycol).

Dermal degradation, followed by imperfect repair, is repeated with each intermittent exposure to UV irradiation, leading to accumulation of solar scarring, which can ultimately result in visible photoaging. Retinoic acid can inhibit the induction of c-Jun protein by UV irradiation, thereby preventing increased matrix metalloproteinase activity and the ensuing dermal damage [1]. Moreover, Lys can raise the moisture and collagen content of mouse dermis by inducing the expression level of collagen and hyaluronic acid [37]. For the present 5-week animal experiments, the recovery rates of photoaged skin were higher in all Ada-treated mice than in the control group at all treatment time points. However, the recovery rate was only improved by 19–33% in the group treated with Ada dissolved in Milli-Q water. More significant improvements were observed in the US + Ada-LysMB group (33–70%) and the US + LysMB + Ada (21–57%) groups. In addition, the morphological changes detected after UV exposure using hematoxylin & eosin and modified VVG staining analyses recapitulated the features of human wrinkled skin, with the segmentation of elastic fibers as well as a decrease in collagen density.

Interestingly, these changes were ameliorated significantly in mice treated with US + Ada-LysMBs or US + LysMBs +Ada. Therefore, the combined effects of Ada and Lys in our experiments might boost the anti-photoaging effects of Ada and repair UV-induced wrinkles. However, their molecular mechanism in various signaling pathways of photoaging [38] warrants further experimental work.

In summary, we demonstrated that the novel US-assisted Ada-LysMBs could improve the photodamaged skin in UV-irradiated mice. Ada-LysMBs were assembled by electrical adsorption of Ada onto LysMBs and were stable in Milli-Q water. Ada loading was proportional to its concentration, and loading on LysMBs enabled the use of a lower but still effective concentration with negligible irritation. The characteristic of higher release at pH 7.4 than at pH 5.5 could be used for controlled-release delivery during the pH alteration from the outer to the inner epidermis. Our findings suggest a new, promising approach for the treatment of photoaging.

## Supporting information

S1 Fig Chemical structure of adapalene. (TIF)

S2 Fig Scheme of the adapalene-coated lysozyme-shelled microbubbles may be delivered via the cracking surface and the dissociated adapalene and lysozyme in deeper skin may enable recovery of photodamaged skin. (TIF)

S3 Fig Scheme of in vitro release study by sonication and dialysis. (TIF)

S4 Fig Scheme of in vitro skin penetration by using static Franz diffusion cells. (TIF)

S5 Fig. Schematic diagram of in vivo animal UVA treatment (A) and photograph of the system used for UVA treatment (B) (TIF)

S6 Fig Schematic illustration of the animal experimental procedure. (TIF)

S7 Fig Schematic diagram of wrinkle measurement processing from image to the numbers of pixels. (TIF)

S1 File Additional information of animal study. (PDF)

S1 Checklist The ARRIVE guidelines checklist. (PDF)

S1 Data (XLSX)

S1 Table (XLSX)

## References

[1] Fisher GJ, Voorhees JJ. Molecular mechanisms of photoaging and its prevention by retinoic acid: ultraviolet irradiation induces MAP kinase signal transduction cascades that induce Ap-1-regulated matrix metalloproteinases that degrade human skin in vivo. J. Investig. Dermatol. Symp. Proc. 1998; 3:61–68. 9732061

[2] Casale C, Imparato G, Urciuolo F, Rescigno F, Scamardella S, Escolino M, et al Engineering a human skin equivalent to study dermis remodeling and epidermis senescence in vitro after UVA exposure. J. Tissue Eng. Regen. Med. 2018; 12:1658–1669. 10.1002/term.2693 29763974

[3] Green LJ, McCormick A, Weinstein GD. Photoaging and the skin. The effects of tretinoin. Dermatol. Clin. 1993; 11:97–105. 8435921

[4] Izumi AK, Marples RR, Kligman AM. Senile (solar) comedones. J. Invest. Dermatol. 1973; 61:46–50. 10.1111/1523-1747.ep12674145 4268867

[5] Mukherjee S, Date A, Patravale V, Korting HC, Roeder A, Weindl G. Retinoids in the treatment of skin aging: an overview of clinical efficacy and safety. Clin. Interv. Aging. 2006; 1:327–348. 10.2147/ciia.2006.1.4.327 18046911 PMC2699641

[6] Biniek K, Levi K, Dauskardt RH. Solar UV radiation reduces the barrier function of human skin. Proc. Natl. Acad. Sci. U.S.A. 2012; 109:17111–17116. 10.1073/pnas.1206851109 23027968 PMC3479513

[7] Yaar M, Gilchrest BA. Photoageing: mechanism prevention and therapy. Br. J. Dermatol. 2007; 157:874–887. 10.1111/j.1365-2133.2007.08108.x 17711532

[8] Balkrishnan R, McMichael AJ, Hu JY, Camacho FT, Shew KR, Bouloc A, et al Correlates of health-related quality of life in women with severe facial blemishes. Int. J. Dermatol. 2006; 45:111–115. doi: 10.1111/j.1365-4632. 2004.02371.x 10.1111/j.1365-4632.2004.02371.x 16445498

[9] Bae YC, Bae EJ, Wang JH, Gilchrest BA. Changes in Self-Perceptions of Photoaging Severity and Skin Cancer Risk After Objective Facial Skin Quality Analysis. J. Drugs, Dermatol. 2017; 16:453–459. doi: S1545961617P0453X 28628681

[10] Kligman LH. Effects of all-trans-retinoic acid on the dermis of hairless mice. J. Am. Acad. Dermatol. 1986; 15:779–785, 884–777. 10.1016/s0190-9622(86)70234-x 3771852

[11] Kligman LH, Duo CH, Kligman AM. Topical retinoic acid enhances the repair of ultraviolet damaged dermal connective tissue. Connect. Tissue Res. 1984; 12:139–150. 10.3109/03008208408992779 6723309

[12] Herane MI, Orlandi C, Zegpi E, Valdes P, Ancic X. Clinical efficacy of adapalene (differin) 0.3% gel in Chilean women with cutaneous photoaging. J. Dermatolog. Treat. 2012; 23:57–64. 10.3109/09546634.2011.631981 22007702

[13] Kang S, Goldfarb MT, Weiss JS, Metz RD, Hamilton TA, Voorhees JJ, et al Assessment of adapalene gel for the treatment of actinic keratoses and lentigines: a randomized trial. J. Am. Acad. Dermatol. 2003; 49 83–90. 10.1067/mjd.2003.451 12833014

[14] Kligman LH. The hairless mouse and photoaging. Photochem. Photobiol. 1991; 54:1109–1118. 10.1111/j.1751-1097.1991.tb02134.x 1775532

[15] Leyden J J. Topical treatment of acne vulgaris: retinoids and cutaneous irritation. J. Am. Acad. Dermatol. 1998; 38(4):S1–4. doi:S0190-9622(98)70138-0 10.1016/s0190-9622(98)70138-0 9555819

[16] Bhalekar M, Upadhaya P, Madgulkar A. Formulation and evaluation of Adapalene-loaded nanoparticles for epidermal localization. Drug Deliv. Transl. Res. 2015; 5:585–595. 10.1007/s13346-015-0261-z 26483036

[17] Liao AH, Hung CR, Lin CF, Lin YC, Chen HK. Treatment effects of lysozyme-shelled microbubbles and ultrasound in inflammatory skin disease. Sci. Rep. 2017; 7 41325 10.1038/srep41325 28117399 PMC5259758

[18] Liao AH, Lu YJ, Lin YC, Chen HK, Sytwu HK, Wang CH. Effectiveness of a Layer-by-Layer Microbubbles-Based Delivery System for Applying Minoxidil to Enhance Hair Growth. Theranostics. 2016a; 6: 817–827. 10.7150/thno.14932 27162552 PMC4860890

[19] Liao AH, Ma WC, Wang CH, Yeh MK. Penetration depth concentration and efficiency of transdermal alpha-arbutin delivery after ultrasound treatment with albumin-shelled microbubbles in mice. Drug Deliv. 2016b, 23:2173–2182. 10.3109/10717544.2014.951102 25148541

[20] Park D, Ryu H, Kim HS, Kim YS, Choi KS, Park H, et al Sonophoresis using ultrasound contrast agents for transdermal drug delivery: an in vivo experimental study. Ultrasound Med. Biol. 2012; 38:642–650. 10.1016/j.ultrasmedbio.2011.12.015 22341597

[21] Niyonsaba F, Ogawa H. Protective roles of the skin against infection: implication of naturally occurring human antimicrobial agents beta-defensins cathelicidin LL-37 and lysozyme. J. Dermatol. Sci. 2005; 40:157–168. 10.1016/j.jdermsci.2005.07.009 16150577

[22] Seite S, Zucchi H, Septier D, Igondjo-Tchen S, Senni K, Godeau. Elastin changes during chronological and photo-ageing: the important role of lysozyme. J. Eur. Acad. Dermatol. Venereol. 2006; 20:980–987. 10.1111/j.1468-3083.2006.01706.x 16922949

[23] Han YH, Zhang ZW, Su J, Zhang B, Li S, Xu SW. Effects of chicken selenoprotein W on H_2_O_2_-induced apoptosis in CHO-K1 cells. Biol. Trace Elem. Res. 2012; 147:395–402. 10.1007/s12011-011-9311-7 22207219

[24] Harde H, Agrawal AK, Katariya M, Kale D, Jain S. Development of a topical adapalene-solid lipid nanoparticle loaded gel with enhanced efficacy and improved skin tolerability. RSC Av. 2015; 5:43917–43929.

[25] Erdem CE, Sankur B, Tekalp AM. Performance measures for video object segmentation and tracking. IEEE Trans. Image Process. 2004; 13: 937–951. 10.1109/tip.2004.828427 15648860

[26] Ahammer H, DeVaney TT. The influence of edge detection algorithms on the estimation of the fractal dimension of binary digital images. Chaos. 2004; 14:183–188. 10.1063/1.1638947 15003059

[27] Gao J, Wang Z, Liu H, Wang L, Huang G. Liposome encapsulated of temozolomide for the treatment of glioma tumor: preparation, characterization and evaluation. Drug Discov. Ther. 2015; 9:205–212. 10.5582/ddt.2015.01016 26193943

[28] Fan Y, Jeong JH, You GY, Park JU, Choi TH, Kim S. An Experimental Model Design for Photoaging. J. Craniofac. Surg. 2015; 26:e467–471. 10.1097/SCS.0000000000001902 26267568

[29] Hung CF, Fang CL, Al-Suwayeh SA, Yang SY, Fang JY. Evaluation of drug and sunscreen permeation via skin irradiated with UVA and UVB: Comparisons of normal skin and chronologically aged skin. J Dermatol Sci 2012; 68: 135–148. 10.1016/j.jdermsci.2012.09.005 23026054

[30] Sezgin M, Sankur B. Survey over image thresholding techniques and quantitative performance evaluation. J Elecron Imaging 2004;13: 146–168.

[31] Canny J. A computational approach to edge detection. IEEE Trans Pattern Anal Mach Intell 1986;8: 679–698. 21869365

[32] Yildirim S, Gurel MS, Gungor S, Tekeli O, Canat D. Comparison of efficacy of chemical peeling with 25% trichloroacetic acid and 0.1% retinoic acid for facial rejuvenation. Postepy Dermatol Alergol. 2016; 33:199−205. 10.5114/ada.2016.60612. 27512355 PMC4969415

[33] Imoto T, Johnson LN, North ACT, Phillips DC, Rupley JR. Enzymes, 3rd ed. New York, NY, USA: Academic Press; 1972 p. 665–868.

[34] Narambuena CF, Longo GS, Szleifer I. Lysozyme adsorption in pH-responsive hydrogel thin-films: the non-trivial role of acid-base equilibrium. Soft Matter 2015;11: 6669–6679. 10.1039/c5sm00980d 26219383

[35] Diarrassouba F, Remondetto G, Garrait G, Alvarez P, Beyssac E, Subirade M. Self-assembly of β-lactoglobulin and egg white lysozyme as a potential carrier for nutraceuticals. Food Chem 2015;173: 203–209. 10.1016/j.foodchem.2014.10.009 25466013

[36] Turner NG, Cullander C, Guy RH. Determination of the pH gradient across the stratum corneum. J Investig Dermatol Symp Proc 1998;3: 110–113. 10.1038/jidsymp.1998.23 9734823

[37] Han HB, Li X, Yu K, Ma WZ, Cao ZC, An GF, et al Egg white lysozyme promoted collagen secreting of dermal fibroblasts in mice. Asian J Anim Vet Adv 2011;6: 667–677.

[38] Kaur A, Thatai P, Sapra B. Need of UV protection and evaluation of efficacy of sunscreens. J Cosmet Sci. 2014; 65315–345. 25682622

