# Supplementary figures and images for: Application of ultrasound-mediated adapalene-coated lysozyme-shelled microbubbles in UVA-induced skin photoaging
Source: PLoS One. 2020 May 21;15(5):e0232617. doi: 10.1371/journal.pone.0232617 (PMC7242023; doi:10.1371/journal.pone.0232617)

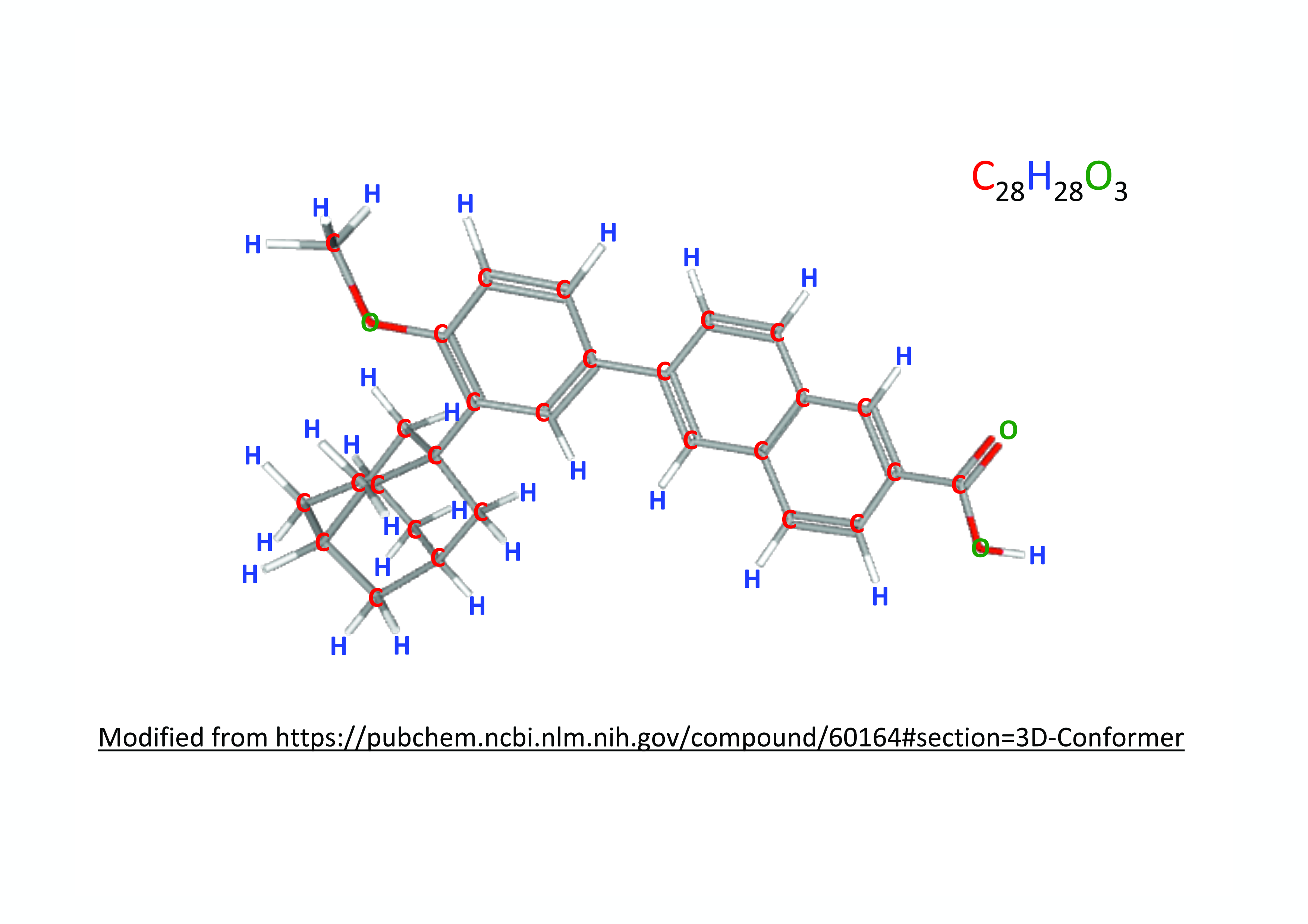

Supplement: S1 Fig — (TIF) [file pone.0232617.s001.tif]

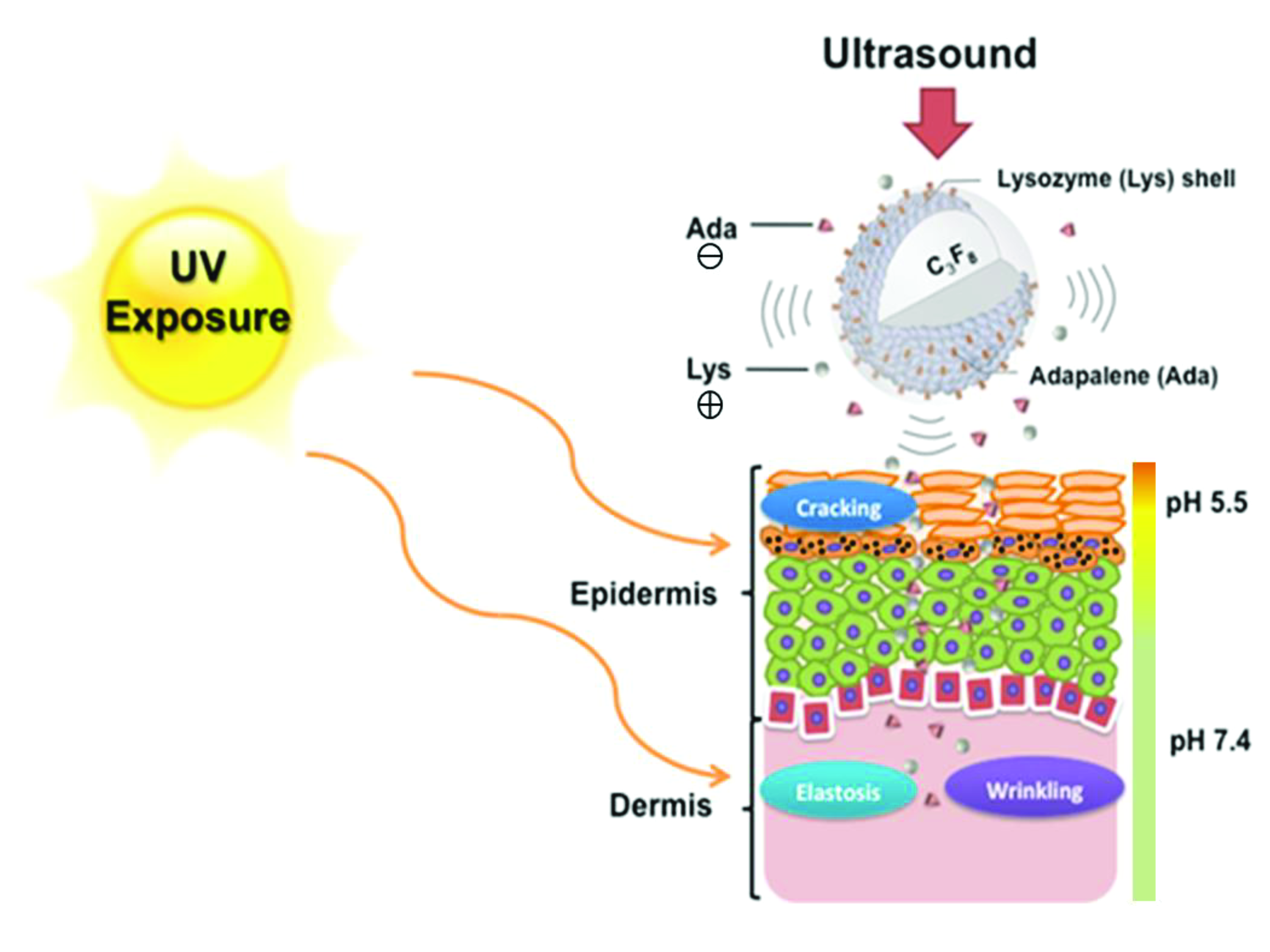

Supplement: S2 Fig — (TIF) [file pone.0232617.s002.tif]

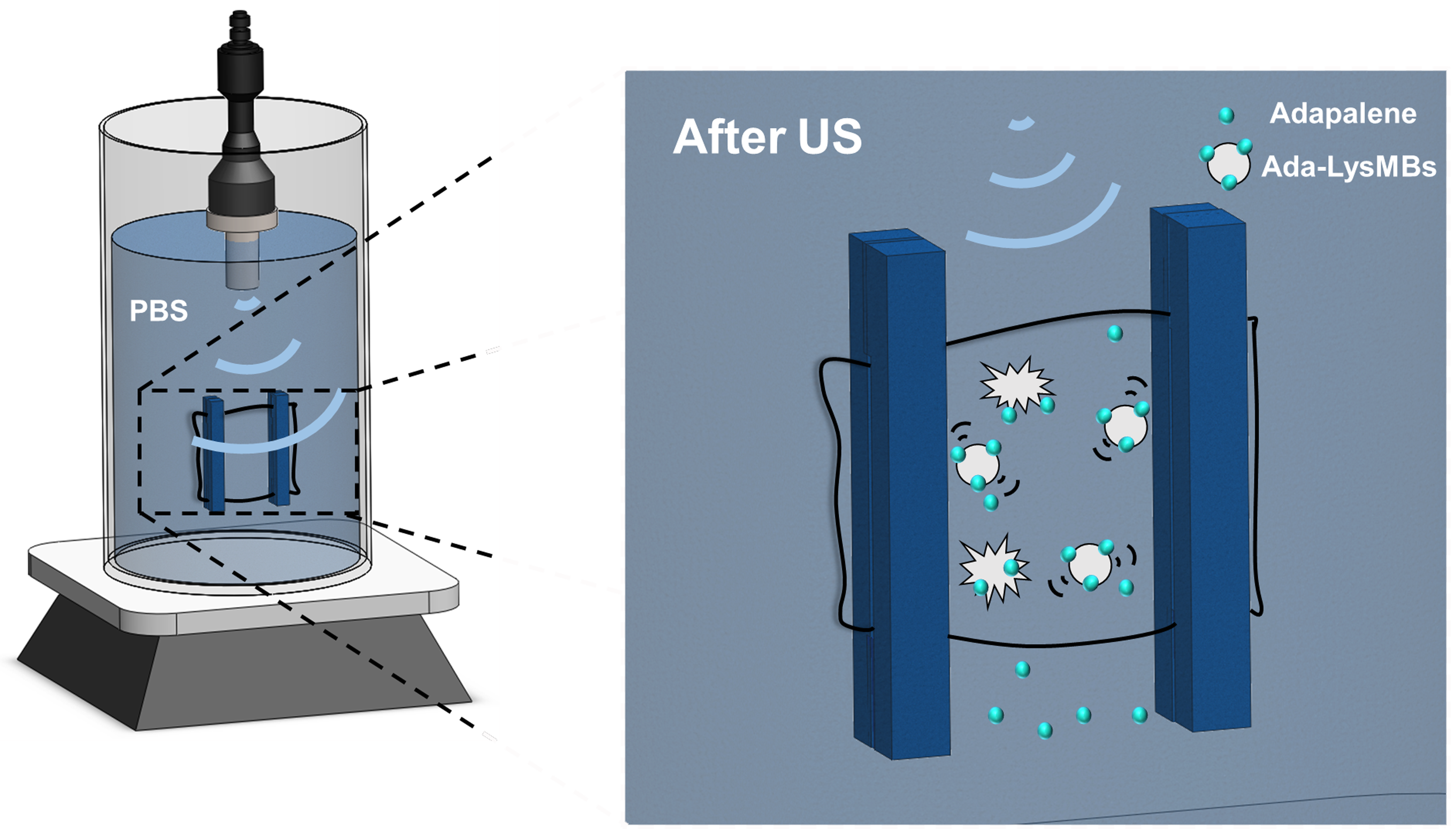

Supplement: S3 Fig — (TIF) [file pone.0232617.s003.tif]

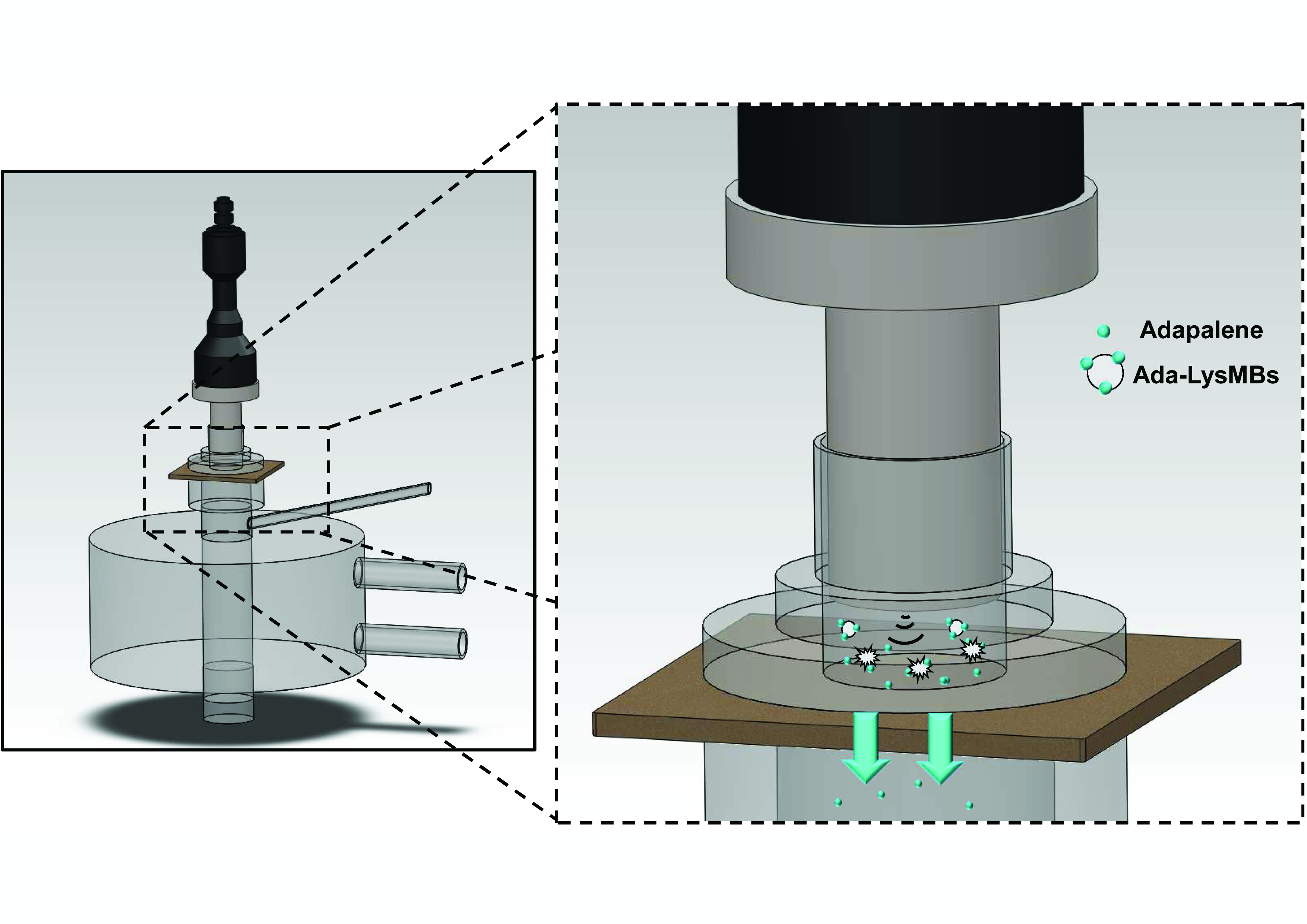

Supplement: S4 Fig — (TIF) [file pone.0232617.s004.tif]

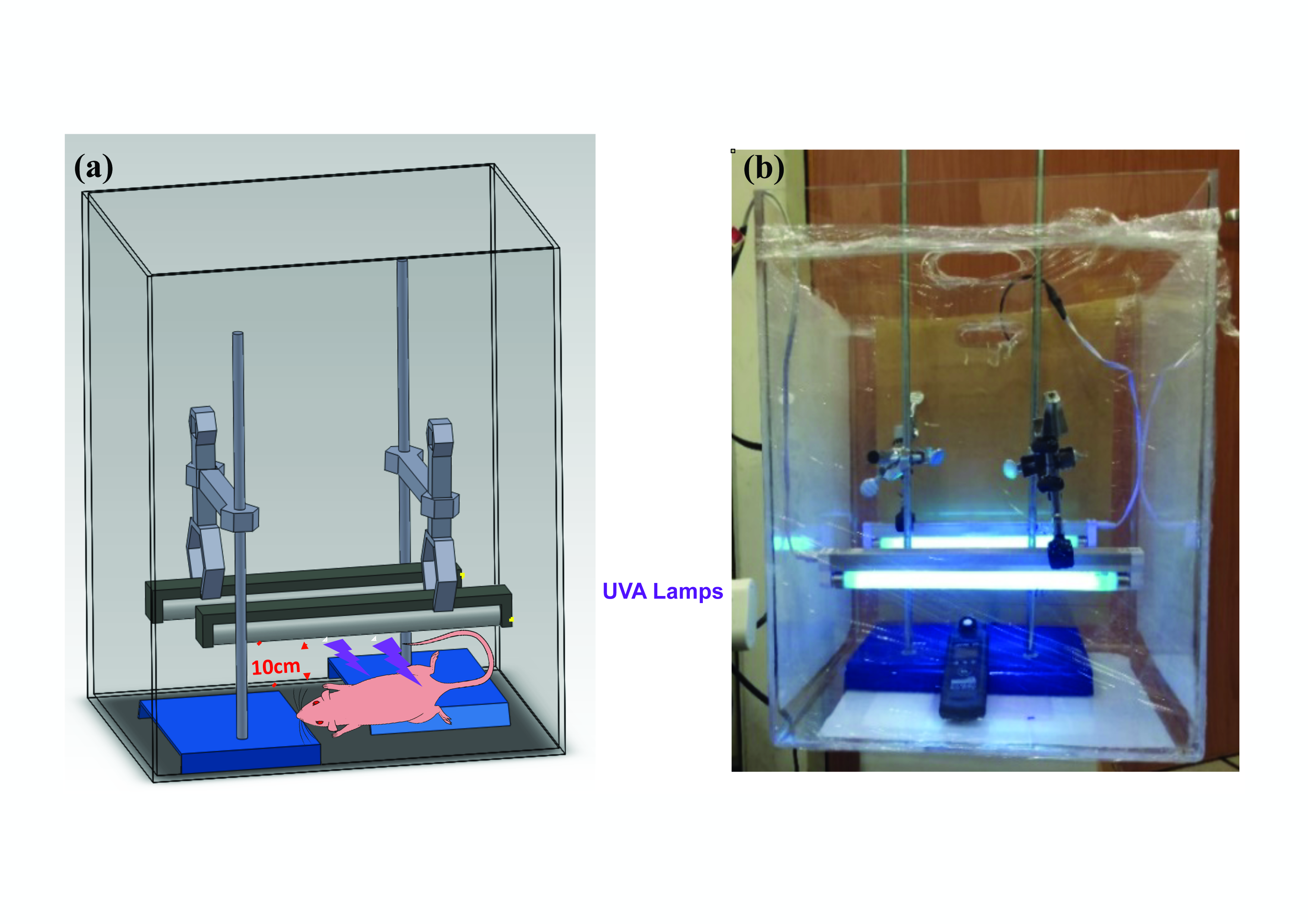

Supplement: S5 Fig. Schematic diagram of in vivo animal UVA treatment (A) and photograph of the system used for UVA treatment (B) — (TIF) [file pone.0232617.s005.tif]

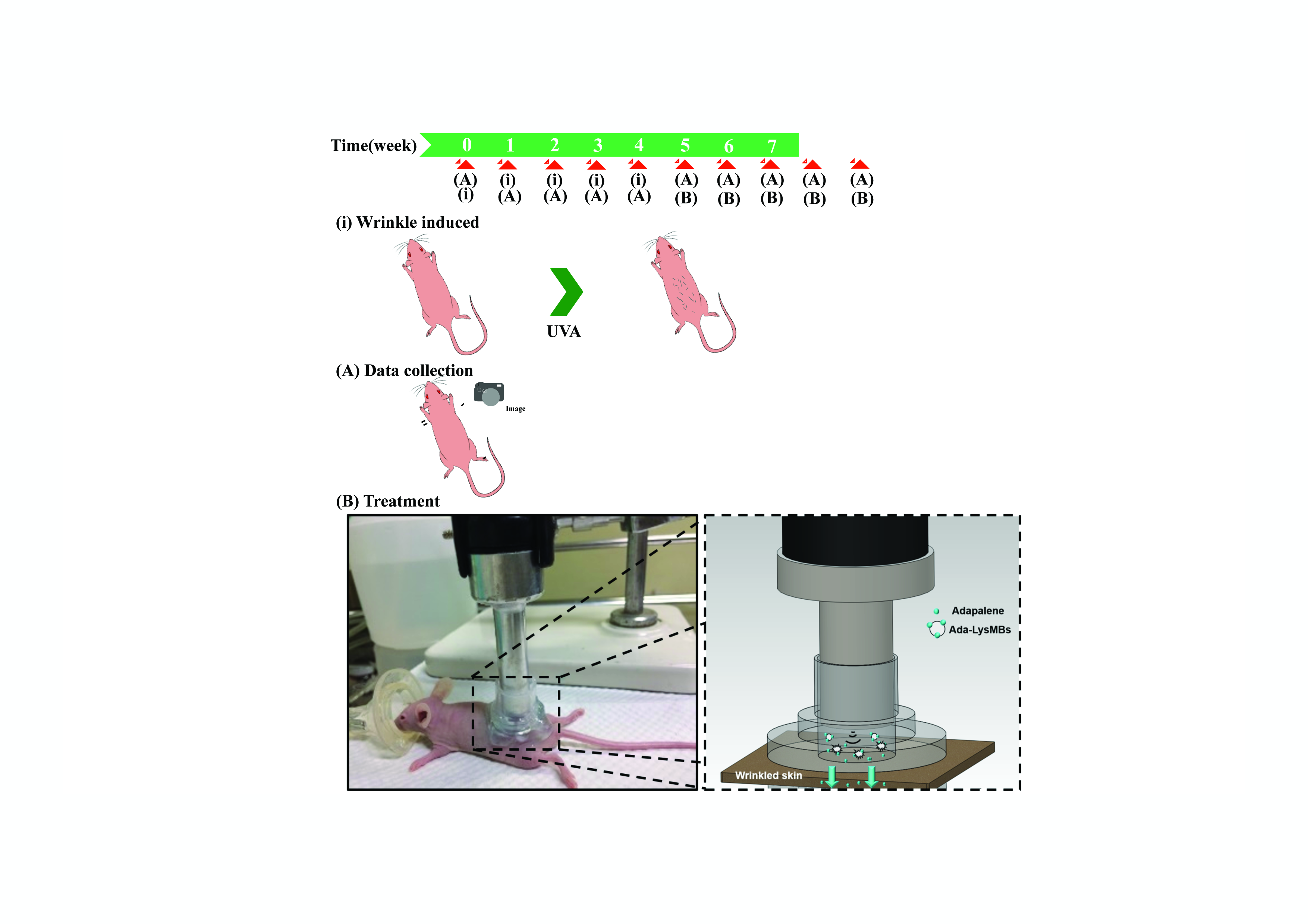

Supplement: S6 Fig — (TIF) [file pone.0232617.s006.tif]

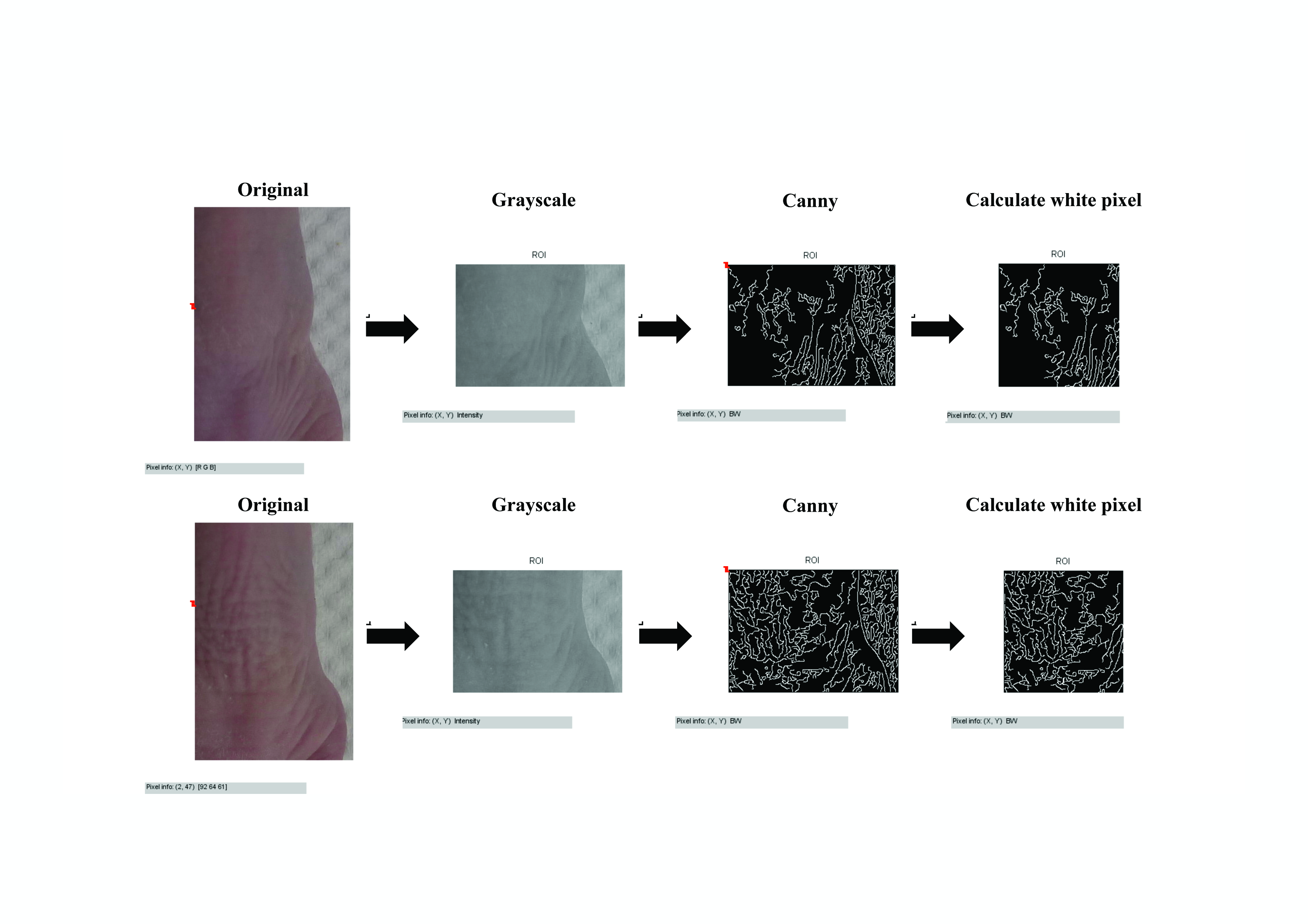

Supplement: S7 Fig — (TIF) [file pone.0232617.s007.tif]
