# Supplementary material for: Application of ultrasound-mediated adapalene-coated lysozyme-shelled microbubbles in UVA-induced skin photoaging
Source: PLoS One. 2020 May 21;15(5):e0232617. doi: 10.1371/journal.pone.0232617 (PMC7242023; doi:10.1371/journal.pone.0232617)
Supplement: S1 File — (PDF) [file pone.0232617.s008.pdf]

國防醫學院實驗動物照護及使用委員會
動物實驗核准證明書

核發日期：2016年12月8日

下列研究計畫業經本學院實驗動物照護及使用委員會（IACUC）審查通過，准予該計畫依申請內容執行動物實驗。計畫主持人應對申請表內容全權負責；該計畫主持人及所屬研究人員應遵守相關法令及本學院各項動物管理代養相關規定，並顧及取代、減量及精緻化等3R原則。

動物實驗核准編號：IACUC-16-328

計畫名稱：超音波微泡系統輔助微創手術診斷治療之應用

計畫主持人：廖愛禾

合作主持人：無

本證明書有效期間：2017年1月2日 至 2017年12月31日

國防醫學院實驗動物照護及使用委員會
NDMC IACUC
National Defense Medical Center Institutional Animal Care and Use Committee

執行秘書	首席獸醫師	召集人
邱俊龍	方美佐	馬國興
